# Bioinformatic Design of Dendritic Cell-Specific Synthetic Promoters

**DOI:** 10.1021/acssynbio.2c00027

**Published:** 2022-04-07

**Authors:** Abayomi O. Johnson, Susan B. Fowler, Carl I. Webster, Adam J. Brown, David C. James

**Affiliations:** † Department of Chemical and Biological Engineering, University of Sheffield, Mappin Street, Sheffield S1 3JD, U.K.; ‡ SynGenSys Limited, Freeths LLP, Norfolk Street, Sheffield S1 2JE, U.K.; § Antibody Discovery and Protein Engineering, R&D, AstraZeneca, Cambridge CB21 6GH, U.K.; ∥ Discovery Sciences, R&D, AstraZeneca, Cambridge CB21 6GH, U.K.

**Keywords:** dendritic cells, synthetic promoter, transcriptional factors, promoter architecture, genome mining

## Abstract

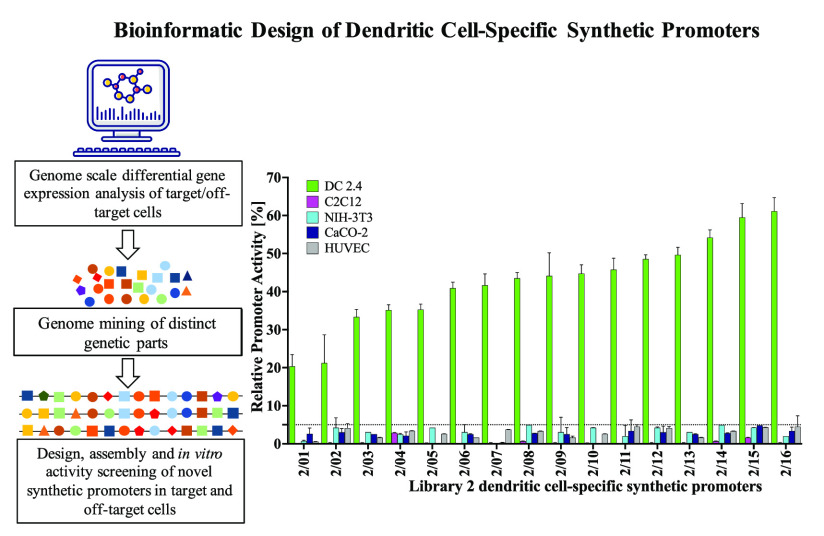

Next-generation DNA vectors for cancer immunotherapies and vaccine development require promoters eliciting predefined transcriptional activities specific to target cell types, such as dendritic cells (DCs), which underpin immune response. In this study, we describe the *de novo* design of DC-specific synthetic promoters *via in silico* assembly of *cis*-transcription factor response elements (TFREs) that harness the DC transcriptional landscape. Using computational genome mining approaches, candidate TFREs were identified within promoter sequences of highly expressed DC-specific genes or those exhibiting an upregulated expression during DC maturation. Individual TFREs were then screened *in vitro* in a target DC line and off-target cell lines derived from skeletal muscle, fibroblast, epithelial, and endothelial cells using homotypic (TFRE repeats in series) reporter constructs. Based on these data, a library of heterotypic promoter assemblies varying in the TFRE composition, copy number, and sequential arrangement was constructed and tested *in vitro* to identify DC-specific promoters. Analysis of the transcriptional activity and specificity of these promoters unraveled underlying design rules, primarily TFRE composition, which govern the DC-specific synthetic promoter activity. Using these design rules, a second library of exclusively DC-specific promoters exhibiting varied transcriptional activities was generated. All DC-specific synthetic promoter assemblies exhibited >5-fold activity in the target DC line relative to off-target cell lines, with transcriptional activities ranging from 8 to 67% of the nonspecific human cytomegalovirus (hCMV-IE1) promoter. We show that bioinformatic analysis of a mammalian cell transcriptional landscape is an effective strategy for *de novo* design of cell-type-specific synthetic promoters with precisely controllable transcriptional activities.

## Introduction

Dendritic cells (DCs) are a heterogeneous population of professional antigen-presenting cells (APCs) equipped to process protein antigens into peptides which are displayed on the cell surface. This is achieved using major histocompatibility complexes I and/or II as DCs mature and migrate to lymphoid tissues to initiate an immune response.^1−5^ In particular, monocyte-derived dendritic cells (moDC) and conventional (myeloid) dendritic cells (cDCs) have been identified as crucial nonlymphoid DC subsets for *in vivo* recombinant antigen expression, largely owing to their presence in the skeletal muscle, which is a major site for vaccine delivery.^1,5,6^ In addition, their peculiar migratory capabilities and excellent ability to induce antigen-specific T-cell responses have further solidified their potential for diverse immunotherapeutic applications.^1,7^ Cell type-specific antigen expression in DCs is an underpinning requirement for safe and efficacious DC-based immunotherapies, as antigen expression in the general cell population could be unsafe or at best redundant. Accordingly, *in vivo* antigen expression in DCs has been shown to be achievable by targeting the tropism of viral/nonviral DNA vectors to DCs and/or at the transcriptional level—by using DC-specific promoters to drive antigen expression in vector systems.^8−12^ However, the complex design requirements for physical targeting have hampered the sustainable development of safe and efficacious DNA-based vaccine vectors; therefore, transcriptional targeting remains a critical focal point for the development of vaccines and cancer immunotherapies.^12^

Endogenous enhancers or enhancer fragments of DC-specific genes (genomic regions upstream of DC-specific genes) have previously been deployed to drive DC-specific antigen expression in vaccine vectors. For example, promoters of the dectin-2,^13,14^ DC-STAMP and DC-SIGN,^15^ CD11c,^15,16^ and the actin-bundling fascin gene^17^ have been used to transcriptionally target antigen expression to different DC subsets. However, endogenous promoters are typically encoded on relatively long stretches of DNA, thus limiting the transgene(s) sizes that can be incorporated into DNA vectors. They also display minimal activity compared to the hCMV-IE1 promoter, which displays a relatively nonspecific high level of transcriptional activity (TA) across mammalian cell types.^18^ In addition, endogenous promoters possess a vaguely defined architecture, as they are naturally evolved for a diverse array of genetic functionalities.^19^ Notably, the heterogeneity in transcriptional factor (TF)-binding motif population potentially compromises the specificity of promoter activity, particularly when evaluated in a broader range of cell types. Moreover, cell-type specificity in gene expression is highly subjective, often depending on the base cell types that specificity is compared against. These fundamental drawbacks have significantly limited the repertoire of promoters available for DC-specific antigen expression.

Addressing these inherent challenges requires proper identification, characterization, and standardization of modular DNA-binding motifs, followed by an informed design strategy to assemble the standardized building blocks into fit-for-purpose synthetic promoters. Indeed, the construction of cell-type-specific synthetic promoters *via* concatenation of binding motifs has been previously described for a similar myeloid cell type—macrophages^20,21^ and several nonmyeloid cell/tissue types, for example, skeletal muscle.^22,23^ However, while these studies successfully identified candidate DNA-binding motifs, inadequate characterization and standardization of the “transcriptional power” of each composite binding motif in a synthetic promoter context or architecture hamper the generation of large arrays of functionally relevant synthetic promoters with predefined activity and specificity levels.

Previously, we have demonstrated the construction of cell-type-specific promoters *via* robust *in silico* transcriptomic data analysis of TF expression dynamics^24,25^ and *via* genome-mined overrepresented transcription factor regulatory elements (TFREs) in endogenous promoters of genes of interest in CHO cell lines in a fashion that obviates the need to design and screen large libraries of randomly assembled TFREs.^26,27^ In this study, we have developed an informatics workflow that leverages both approaches to (i) identify modular DC-specific-binding motifs in the endogenous promoters of genes that confer DC-specific TA and (ii) define promoter assembly rules for generating DC-specific synthetic promoters. In particular, we aimed to design DC-synthetic promoters with negligible activity in the bulk of cell types associated with the skeletal muscle tissue—myocytes, fibroblasts in the associated connective tissue, endothelial cells in the surrounding vasculature, and epithelial cells in the surrounding epidermal layer. Accordingly, we utilized the DC2.4 murine monocyte-derived DC line as a model cell line for the *in vitro* assay of transcriptional activities of *in silico* designed promoter constructs based on previous studies that have validated this cell line as a powerful tool for DC research.^7,28−31^ The specificity of promoter activity was evaluated by *in vitro* screening in cell lines C2C12, NIH-3T3, and CaCO-2 as model cell lines for myocytes, fibroblasts, and the epithelium, respectively, and HUVEC primary cells as the model cell type for endothelial cells. Our results demonstrate that analysis of the transcriptional landscape of target cell type(s) and off-target cell types critically underpins *in silico* design of functionally active cell-type specific synthetic promoters.

## Results and Discussion

### *In Silico* Analysis of Candidate DC-Specific Transcriptional Factor Regulatory Elements

Our approach to designing moDC- and cDC-specific synthetic promoters involved the identification of individual TF-binding motifs and cognate endogenous TFs associated with a high constitutive expression of DC-specific genes and/or those which influence upregulation of TA during DC maturation (Table 1). To achieve this, we first queried publicly available transcriptomic data to identify genes with high TA in our target cell of interest. Transcriptomic data of gene expression dynamics in human monocyte-derived DCs and conventional DCs were derived from the ArrayExpress database at EMBL-EBI (www.ebi.ac.uk/arrayexpress/experiments/E-MTAB-6192) and NCBI GEO Database (accession GSE101878),^32^ respectively. Accordingly, we ranked the genes from each dataset in order of mRNA quantification, then selected conserved genes exhibiting high mRNA levels across both datasets (Supporting Information, Tables S1 and S2). In order to select for genes with high TA specifically in moDC and cDC from this dataset, we first identified genes expressed specifically in moDC and cDC based on cell/tissue-specific expression potential analysis of the transcriptional data on GeneVestigator V3.^33^ Cell types related to endothelial, epithelial cells, fibroblasts, and skeletal muscle were set as base (off-target) cells, while moDC and cDC were separately set as target cell types (Supporting Information, Table S3). Next, genes common to both the high TA and specificity groups were classed as highly expressed and specific genes in moDC and cDC (Supporting Information, Table S4). Second, we identified genes that are upregulated during the maturation of moDC and cDC based on the available data on DC maturation genetic reprogramming studies by Mahn *et al.* (Supporting Information, Table S5).^34^ All profiled genes were subsequently categorized as follows: (1) A1—highly expressed in moDC, (2) A2—expressed specifically in moDC, (3) A1.2—highly expressed specifically in moDC, (4) B1—highly expressed in cDC, (5) B2—expressed specifically in cDC, (6) B1.2—highly expressed specifically in cDC, (7) C—upregulated in moDC and cDC during maturation (Supporting Information, Table S6).

**Table 1:** Sequence Description of Candidate DC-specific Transcription Factor Response Elements Identified Using a 7-Step Informatics Workflow as Described in Figure 1

TFRE	TFRE sequence	cognate TF(s)	gene source	gene class	start	end	strand
IRF9-A	ctaaaccgagaatcgaaactaagct	IRF9	PML	upregulated	1066	1090	+
c-Rel-A	cttggggtttccaac	c-Rel	SDC4	upregulated	848	862	+
SPI1-A	gtgaaggaggaagtctgaggc	SPI1 (PU.1)	TYROBP	moDC	982	1002	–
IRF4-A	ataggagggctaaagaaagcagaaa	IRF4	FPR3	moDC	942	966	–
JUNB-A	ctcttagtcaccg	JUNB	CCL22	moDC	924	936	–
STAT5A-A	aggtttccgagtattgctt	STAT5A	FCN1	cDC	681	699	+
BATF-A	tcctgactcactg	BATF, JUNB	MRC1	moDC	1	13	+
IRF8-A	ctagattcgaaaccaaaccctgtga	IRF8, IRF4	STAT2	upregulated	21	45	–
RELA-A	gaaggactttccagc	RELA (p65)	NFKBIA	upregulated	756	770	+
IK1-A	catcgggaacacc	IKZF1 (IK1)	CORO1A	cDC	451	463	–
c-Rel-B	gaaggagattccttc	c-Rel	RBBP8	upregulated	454	468	+
SPI1-B	aagcaaggggaagcaggcctc	SPI1	ITGAX	cDC	173	193	–
IRF4-B	gtagatgtggaagtgaaagctacaa	IRF4, SPI1 (PU.1)	CD74	mo/cDC	891	915	–
JUNB-B	gcctgagtcaccg	JUNB	C1orf162	cDC	562	574	–
BATF-B	tcttgactcagtc	BATF, JUNB	CD1A	moDC	718	730	+

From our collection of gene sets of interest, we first aimed to identify TFREs commonly represented within the promoters of each gene class. We hypothesized that these TFREs are critical to the transcriptional phenotype of the genes in each gene class and would be key inputs for a bottom-up approach to designing highly transcriptionally active DC-specific synthetic promoters. Endogenous promoter sequences of all A1.2, B1.2, and C genes were derived from a genomic region upstream of each gene from the Eukaryotic Promoter Database (EPA).^35^ We limited endogenous promoter sequence length to −1000 to +100 relative to the transcriptional start site of the genes of interest in line with common knowledge that this cistrome region is relatively more concentrated with binding motifs which influence gene expression dynamics.^19,36^ As such, the Common TF tool of Gene Regulation software suites of Genomatix (https://www.genomatix.de/) was used for *in silico* analysis of the endogenous promoter sequences to identify common TFREs in the endogenous promoter sequences of genes in each gene class of interest. A 25% threshold of the gene set and matrix settings core similarity (1.0) and matrix similarity (optimized) were applied. On this note, a total of 745 TFRE types common to at least 25% of promoters of each gene class were identified (Supporting Information, Figure S1A).

In order to rapidly curate the literature for TFREs of cell-type-specific TFs within the promoters of each gene class, we analyzed the general TFRE pool of all genes in the three gene classes using MatInspector, Matrix Library 11.2 (http://www.genomatix.de/matinspector.html). We next applied the tissue specificity filter function to identify discrete TFRE types of *trans*-activators specific to APCs, which is the closest available description of DCs that the tool afforded (Supporting Information, Tables S7 and S8). From this *in silico* analysis, 52 TFRE types of TFs unique to APCs were identified (Supporting Information, Figure S1B). In total, ∼800 TFRE types (spanning ∼2000 unique TFRE sequences) were identified from *in silico* endogenous promoter analysis.

### Identification of DC-Specific TFs and TFs Responsible for Gene Upregulation during DC Maturation

While the transcriptional phenotype of our profiled genes of interest is conferred by discrete TFRE sequences in endogenous promoter sequences, it is, in fact, the relative presence of cognate TFs binding to TFREs in DCs that directly actuates these transcriptional phenotypes. Therefore, we used a comprehensive analysis of the DC-specific TF repertoire as a second vital input to select DC-specific candidate TFREs from endogenous promoter sequences. Although the relative abundance of any given TF in DCs does not necessarily connote DC-specific TA of cognate TFREs, it is an important initial criterion for the selection of useful *trans*-elements. Moreover, it may be argued that to construct a heterologous synthetic circuitry, it is advantageous to harness the transcriptional power of highly abundant TFs in order to ensure that TFs also vital to cell maintenance functions are not titrated away from the endogenous *cis*-elements.^24^

**Figure 1:**
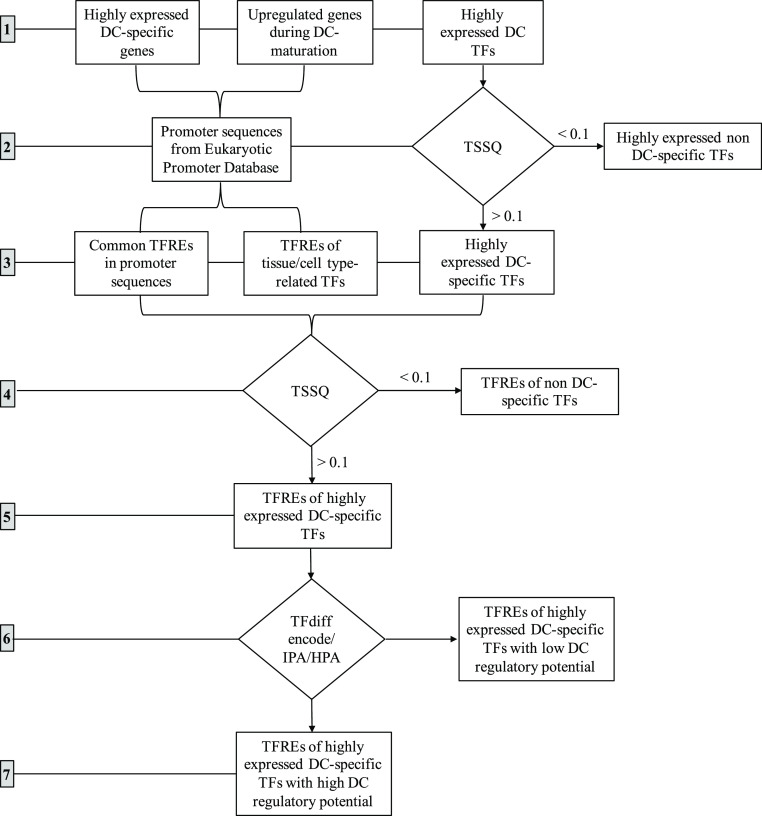
Schematic describing a 7-step informatics workflow to identify TFREs (TF binding sites) for *in silico* design of DC-specific promoters: (1) identification of highly expressed DC-specific genes, upregulated genes during DC maturation, and highly expressed TFs in DCs (monocyte-derived DCs, conventional DCs, and activated conventional DCs); (2) extraction of promoter sequences from the eukaryotic promoter database and elimination of highly expressed non-DC specific TFs; (3) identification of common TFREs in promoter sequences, TFREs of TFs which are peculiar to APCs, and highly expressed DC-specific TFs using the target score specificity quotient (TSSQ) metric; (4) elimination of TFREs of non-DC specific TFs using the TSSQ metric; (5) selection of TFREs of high expressed DC-specific TFs, (6) elimination of TFREs of TF with low regulatory potential in DCs using TFdiff encode, Human Protein Atlas (HPA), and Ingenuity Pathway Analysis (IPA) as sources of information; (7) selection of TFREs of TFs with high regulatory potential in DCs.

To this end, we queried existing literature on cell type-specific TF expression profile from D’Alessio *et al.* to identify TFs specific to DC cell types of interest (Figure 1).^37^ We used the specificity score (SS) parameter (1–1055), which ranks cell type TFs in a decreasing order of abundance, to identify TFs which were not only highly abundant in the moDC, myeloid DC, and activated myeloid DC but also relatively less abundant in the preselected off-target cell types combined (Supporting Information, Table S9). Accordingly, the top ∼10% of highly expressed TFs (top 100 TFs with SS: 1–100) in (i) the moDC, (ii) myeloid or conventional DC, and (iii) activated myeloid DC were selected from this study, cumulating in 170 candidate TFs from all 3 DC types (Supporting Information, Figure S1C). Non-DC specific TFs, which were also among the top ∼10% of highly expressed TFs (TFs with SS: 1–100) in the key 8 off-target cell types predominant in off-target tissue sites, were excluded, leaving a total of 79 DC-specific TF candidates (Supporting Information, Figure S1D). The mean SS values of each TF in all three target cell types (MTSS) and the corresponding mean SS value in all eight off-target cell types (MBSS) were also calculated, thus generating discrete SS values for each TF in target and off-target cell types. In order to rank all 79 candidate TFs in the decreasing order of specific abundance in our DC subsets of interest relative to off-target cell types, we devised a TF specificity metric—TSSQ, which is the normalized difference between the mean SS of each TF in the off-target and target cell types.
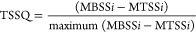
1that is, TSSQ is the difference between MBSS and MTSS values of a given TF *i* of the 79 TF candidates normalized by the highest difference between MBSS and MTSS value of all 79 TFs.

TFs with a TSSQ < 0.1 were eliminated, leaving 69 core DC-specific TF candidates. In addition, all initially excluded non-DC-specific TFs from the list of 170 TF all also had TSSQ values <0.1 (Figure 1, Supporting Information, Figure S2A).

### Selection of DC-Specific TFREs for DC-Specific Promoter Construction

Having identified common *cis*-regulatory elements, DC-related *cis*-elements from endogenous promoters of our genes of interest, and DC-specific *trans*-elements, we proceeded to select cognate TFRE elements of DC-specific TFs, which confer high TA (Figure 1). First, TFRE types of TFs that are common to at least two of the: (i) 745 TFRE types from common TFs analysis, (ii) 52 TFRE types of APC from MatInspector, (iii) TFREs of the 69 DC-specific TF candidates, were pooled (Supporting Information Figure S2B). From this selection, TFRE types (spanning 44 TFs) common to at least two of these groups were identified (Supporting Information, Figure S2B). Of these 44 TFs, only 25 TFs with a TSSQ > 1 were selected. All 25 TFs of the identified TFREs were classed according to the TSSQ values as 11 class I TFs (TSSQ: 1–0.5) and 14 class II TFs (TSSQ: 0.499–0.100). Two TFs, which were not among the initially identified 69 TFs but which had the highest TSSQ (IRF9 = 0.52, JUNB = 0.46) among TFs identified from *in silico* promoter analysis with common TFs and APC-related, were among the list of 25 TFs. Also, 46 class III TFs, which were among the initial 69 TFs but were not common to TF identified from common TF analysis and/or APC-related TFREs, were excluded from the pool of 69 TFs.

To improve the stringency of our selection of *trans*-elements, we further screened the 25 TF candidates to identify (i) high-transactivation potential TFs using TFdiff encode (*p* value cut off: 0.05), (ii) TFs involved in the transcriptional network of genes upregulated during DC maturation, using IPA, (iii) nucleoplasm-localized *trans*-activators of DC function, identity, or phenotype, using the HPA as a credible source of information (Figure 1). Finally, we selected only TFs common to at least two of the TF groups above and limited the maximum number of TFs per TF family to four in order to achieve heterogeneity across TF families for DC-specific promoter construction. This resulted in a final selection of TFREs of 7/10 TFs from class I: (IRF8, SPI1, IRF4, BATF, c-Rel, STAT5A, IRF9) and 3/13 TFs from class II (IKZF1, RELA, JUNB). Hence, the corresponding TFRE sequences of the 10 selected TFs (∼40 TFRE sequences per TF) formed the TFRE collective for *in silico* design of DC-specific synthetic promoters (Supporting Information, Figure S2C). Notably, the mean of the mean SS of all 10 TFs in the 3 target cell types (MTSS) was ∼4-fold lower than the mean of their mean SS in all 8 off-target cell types (MBSS), thus suggesting that all 10 TFs were indeed unique to DCs (*p* value < 0.0001, two-tailed) (Supporting Information, Figure S2D).

### *In Vitro* Construction and Screening of Homotypic Promoters

**Figure 2:**
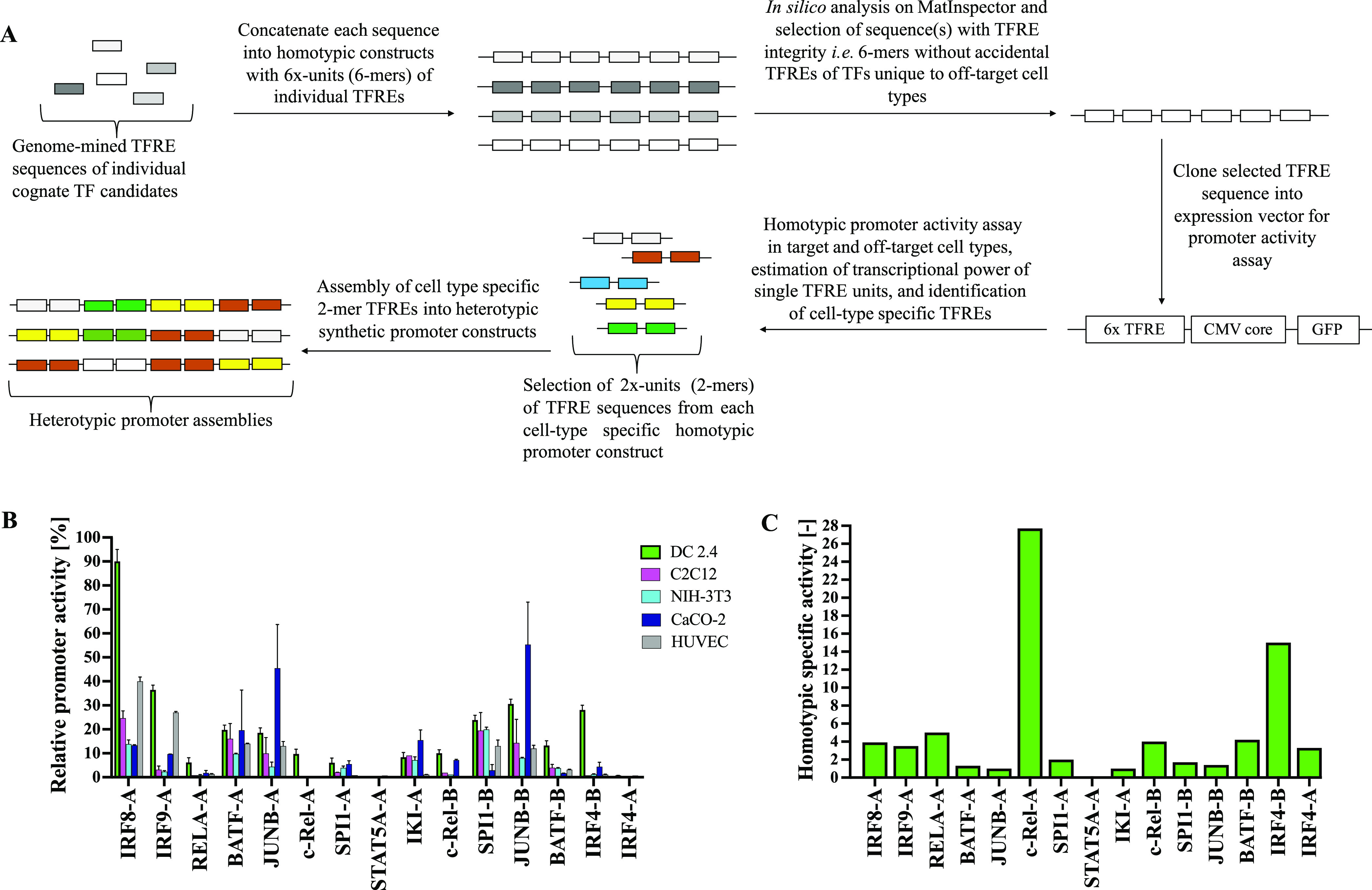
*In vitro* construction and screening of homotypic constructs of TFREs with respect to cell-type specificity and bioactivity for heterotypic synthetic promoter assembly. (A) Candidate TFREs derived from an informatics workflow are concatenated into 6-repeat homotypic promoter constructs which are screened for sequence composition on MatInspector. Homotypic assemblies, which do not contain undesired accidental sequences at TFRE–TFRE junctions, are selected and cloned into a GFP-reporter vector possessing the hCMV-IE1 core promoter element upstream of the CDS of the GFP, which are screened *in vitro* in the target (DC 2.4) and off-target cell types (C2C12, NIH-3T3, CaCO-2, HUVEC). The transcriptional power of single TFRE units is estimated, and cell-type-specific TFREs are identified. 2×-repeat elements (2-mer) of each cell-type-specific TFRE are taken from the homotypic constructs and assembled in varying ratios into heterotypic promoter constructs. (B) Relative promoter activity (RPU) of homotypic (6-mer) constructs of selected TFREs when screened for GFP expression relative to the human cytomegalovirus IE1 (hCMV-IE1) promoter (positive control) in target DC 2.4 cells and all four off-target cells. Values represent the mean ± standard deviation across three independent transfections, each performed in triplicate. (C) Homotypic promoter SS is calculated as a ratio of the RPU in DC2.4 cells and the mean RPU in all four off-target cells.

We have previously utilized homotypic promoters comprising 6× repeat (6-mer) of individual candidate TFRE sequences to characterize the transcriptional power of individual TFRE synthetic promoter building blocks prior to being utilized in the construction of fit-for-purpose heterotypic promoters.^24−26^ Accordingly, 6-mer homotypic units were constructed from TFRE sequences of each selected TF (∼40 sequences per TF) and analyzed on MatInspector to identify accidental TFREs at TFRE–TFRE junctions or embedded within individual TFRE sequence units (Figure 2A). Where possible, 2–3 bp spacer sequences were applied at TFRE–TFRE junctions of the homotypic construct in order to eliminate unwanted accidental TFREs formed at TFRE–TFRE junctions. Next, to ensure proper representation of the TA and target-cell specificity of each TFRE in a 6-mer homotypic architecture, we only selected homotypic constructs that did not possess accidental or embedded sequences of off-target TF and/or repressor sites (Figure 2A). However, we did not eliminate homotypic constructs that possessed accidental or embedded sequences of any other among the 10 TF candidates. Moreover, although we did not prescribe the specific endogenous promoter sources from which representative TFRE sequences of cognate TFs were selected, we mostly restricted the selection of TFREs of class I TFs to group A1.2/B1.2 genes and selection of TFREs of TFs involved in DC maturation (*e.g.,* IRF9, c-Rel, and Rel-A) to group C genes (Table 1). As such, we anticipated that this TFRE sequence selection protocol would potentially increase the chance that selected TFRE sequences functionally contribute to the TA of their source endogenous promoter.

Accordingly, all selected homotypic promoter sequences were screened for green fluorescence protein (GFP) expression in DC2.4 cells and all four off-target cells using the hCMV-IE1 promoter in the pVAX1-CMV-GFP vector as a positive control (Figure 2A, Supporting Information, Figures S3 and S4).

All homotypic promoters elicited activities between 0.5 and 90% of the hCMV-IE1 promoter in the pVAX1-CMV-GFP vector in DC2.4 cells (Figure 2B). The specificity of the activity of the homotypic promoters (known here as the homotypic promoter SS) was calculated as a ratio of the RPU in DC2.4 cells to the mean RPU of TFREs in all four off-target cell types.
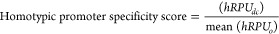
2where *hRPU*_*dc*_ is the RPU of the homotypic promoter in DC2.4 cells, and *hRPU*_*o*_ is the RPU in an off-target cell type.

TFREs c-Rel-A, IRF4-B, c-Rel-B, BATF-B, and RELA-A had the highest homotypic promoter SS (>4), followed by moderate values for IRF9-A, IRF8-A, IRF4-A, and SPI1-A (2–4), while IKI-A, STAT5A-A, JUNB-A, JUNB-B, BATF-A, and SPI1-B had the lowest homotypic promoter SS (<2) (Figure 2C).

### *In Vitro* Construction and Screening of DC-Specific Heterotypic Promoters

Cell-type-specific endogenous promoter activity is a complex cellular phenotype influenced by the binding interaction of specific *trans*-elements (*i.e.,* TFs) with unique genetic signatures comprising distinct *cis*-elements (*i.e.,* TFREs) within endogenous promoters.^19,38^ While this phenotype is naturally encoded in endogenous promoter architectures as complex promoter frameworks/motifs, creating synthetic mimicries of these genetic signatures is a significant challenge. The main interacting criteria for the design of cell-type-specific heterotypic synthetic promoters from distinct TFREs include (i) maintenance of the specificity and TA of individual TFREs proportionately within the heterotypic promoter architecture, (ii) minimization of the accidental bioactive DNA sequence introduction at selected TFRE–TFRE junctions that could compromise promoter specificity and/or activity *via* optimization of the linear arrangement of TFREs.

Based on these core design criteria, we generated, *in silico*, the first library of 42 heterotypic promoters (library 1) using permutations of 12 active TFREs comprising at least 3 different TFRE sequences per promoter in order to create heterogeneity in the endogenous TFs used to drive transcription from synthetic constructs. Furthermore, a number of sequence-specific design elements were employed, including

(i) utilization of 2-mer TFRE units as paired blocks of adjacent single TFRE units. This allowed for a better functional modular coverage of the activity and specificity of selected homotypic constructs in a heterotypic environment (Figure 2A). We postulated that concatenation of 2-mer TFRE units extracted from the homotypic constructs, as opposed to single TFRE blocks, would ultimately reduce the number of undesired accidental TFREs formed when assembled into heterotypic promoters and enable estimation of the strength and specificity of all *in silico* constructed promoters from the TFRE composition and copy number.
(ii) avoidance of non-DC specific multi-TFRE motifs or colocation of TFREs whose TFs have been shown to cooperatively interact to create a non-DC-specific transcriptional signature in the promoter architecture coupled with colocation of TFREs known to cooperatively signify DC-specific transcriptional signatures as a synthetic mimicry of DC-specific promoter models in endogenous promoters of genes of interest. For example, naturally occurring IRF8/IRF4-SPI1-binding motifs (EICE sequence motifs)^39^ and BATF/JUNB-IRF8/IRF4-binding motifs (AICE sequence motifs)^40^ in some endogenous promoters have been identified as the key influencers of DC-specific expression of downstream genes. Alternatively, non-DC-specific TF–TF interaction, such as between RELA and IRF8, positively influences gene expression in a large array of cell types, including fibroblasts and endothelial cells.^41^
(iii) where required, use of appropriate 2–3 bp spacer units to eliminate unintended TFREs between adjacent 2-mer units of different TFREs. We also ensured that adjacent repeat TFRE sequences were limited only to paired TFRE sequences (2-mer units) in order to avoid the formation of long repeat sequences potentially capable of homologous recombination.
(iv) avoidance of heterotypic constructs overpopulated with low-specificity TFREs (*i.e.,* homotypic specific activity < 2), such as SPI1-B, JUNB-B, IKI-A on the premise of such TFREs, would result in very unspecific heterotypic promoters, particularly where they outnumber the more specific TFREs (Figure 2B,C).

**Figure 3:**
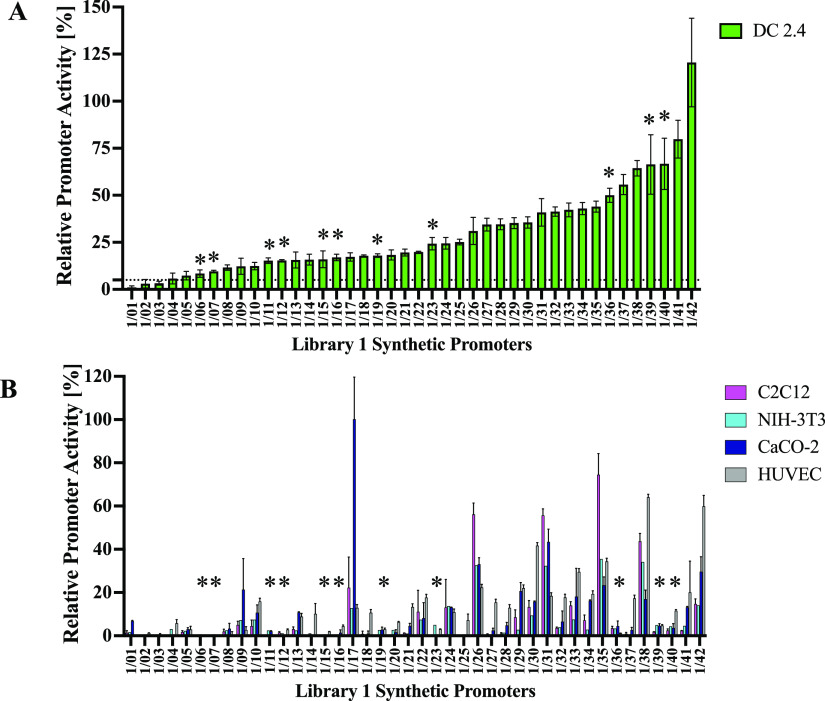
Activity and cell-type specificity of library 1 heterotypic synthetic promoter constructs. Synthetic promoters (42) derived from *in silico* assembly of varying combinations of 2-mer homotypic TFRE blocks were placed upstream of a GFP reporter and transfected into a target DC line (DC 2.4); (A) or alternative off-target human cell lines (C2C12, NIH-3T3, CaCO-2, HUVEC); (B) synthetic promoter activity is shown relative to that observed using the human CMV-IE promoter control construct in each case. 11 synthetic promoters (starred) exhibiting >5% activity (marked by dotted line) in DC2.4 cells and minimal activities in the off-target cells are identified as the most DC-specific first library promoters. Values represent the mean ± standard deviation across three independent transfections, each performed in triplicate.

All 42 first library synthetic promoters consisted of between 6 and 32 TFREs and ranged from 177 to 740 bp in length (Supporting Information Table S10). Next, replacing the control human CMV-IE proximal domain in the pVAX1-CMV-GFP (and retaining the 84 bp hCMV-IE1 core element), all 42 synthetic promoters were cloned upstream of a GFP reporter gene (Figure 2A, Supporting Information, Figure S3) prior to transfection into DC2.4 and four off-target cell types. Transfected cells were then assayed for the cellular GFP content at 24 h post-transfection (Supporting Information, Figure S5A–D). All 42 heterotypic promoters elicited a broad range of activity ranging from ∼1% (1/01) to ∼121% (1/42) relative to the activity in the hCMV-IE1 promoter in pVAX1-CMV-GFP in DC2.4 cells (Figure 3A, Supporting Information, Figure S5A). In the off-target cells, the promoters elicited a different transcriptional output relative to the target DC2.4 cells (Supporting Information, Figure S5B–D). In particular, promoters 1/06, 1/07, 1/11, 1/12, 1/15, 1/16, 1/23, 1/36, 1/39, 1/40, and 1/38 exhibited 8–67% activities (∼8-fold range of activity) relative to the hCMV-IE1 promoter in DC2.4 cells, and low activities in all four off-target cells (Figure 3A,B). These 11 promoters, which exhibit over 5-fold higher activity in the target DC line relative to all off-target cells and <5% RPU in most of the off-target cells, are identified as the most DC-specific first library synthetic promoters (Figure 3A,B, Supporting Information S6). Conversely, promoters such as 1/17 and 1/35 displayed high activities in the off-target cells and low activity in DC2.4 cells (Figure 3A,B).

### TFRE Composition and Unique Transcriptional Behaviour in a Heterotypic Promoter Environment Influences the DC-Specific Activity

In order to delineate the observed activities of the first library of promoters, we first evaluated the homotypic activities of constituent TFREs of each promoter. Based on the assumption that single TFREs account for one-sixth of the strength of respective homotypic promoters, we estimated the heterotypic promoter activity as a summation of the product of the homotypic transcriptional power of each TFRE unit and their respective copy numbers in order to delineate the transcriptional output of all 42 promoters in DC2.4 cells (Figure 2A).
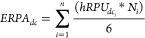
3where *n* is the number of distinct constituent TFRE sequences of any given synthetic promoter, and T_*i*_ is any given TFRE within a given heterotypic promoter, *ERPA*_*dc*_ is the estimated RPU in DC 2.4 cells, and *hRPU*_*dc*_*i*__ is the RPU of a given homotypic promoter of a given TFRE *T*_*i*_ in the target DC 2.4 cell, *N*_*i*_ is the copy number of each TFRE T_*i*_, and *i* is a serial identifier of TFRE.

**Figure 4:**
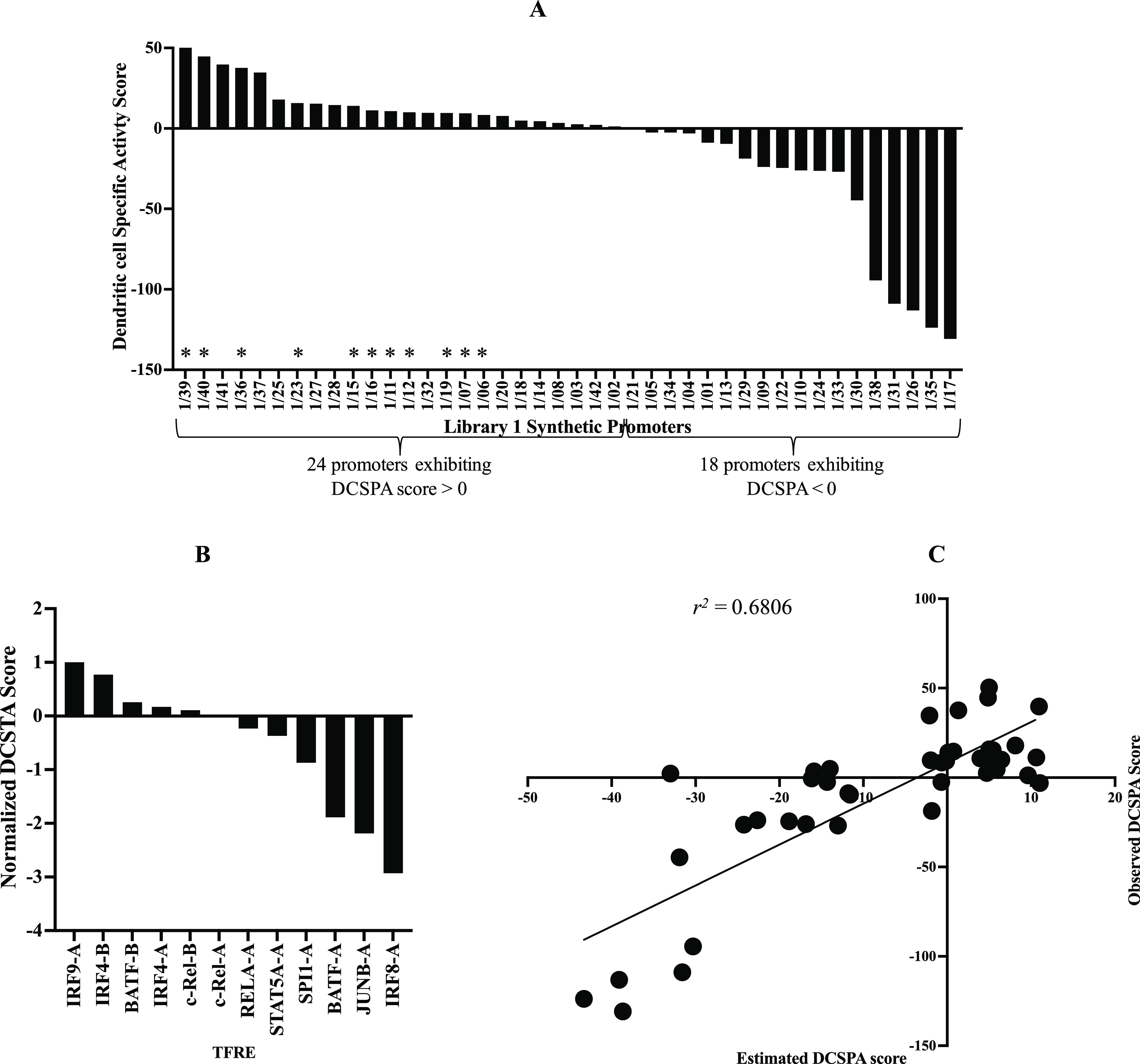
(A) DCSPA score of all 42 first library promoters arranged in descending order of magnitude showing the degree to which each promoter accesses the DC-specific transcriptional landscape. 24 promoters are shown to access the DC-specific transcriptional landscape, and 14 promoters to not access a DC-specific transcriptional landscape. All 11 most DC-specific promoters (starred) exhibit positive DCSPA scores. (B) Normalized DC-specific TFRE activity score of all 12 TFREs arranged in a descending order of magnitude showing the degree of contribution of individual TFREs to DCSPA. (C) Plot of observed DCSPA score and the estimated DCSPA score. A statistically significant relationship (*r*^2^ = 0.6806) is established between the estimated DCSPA score and the observed DCSPA.

However, no significant relationship was established between the estimated RPU and the observed relative promoter activities in DC2.4 cells (*r*^2^ = 0.1952). This indicated a contrast between the transcriptional behavior exhibited by constituent TFRE motifs in a heterotypic environment compared and their respective homotypic promoter activities. Second, we quantified the degree to which each of the 42 promoters specifically harnesses the DC-transcriptional landscape to actuate transcription. This metric—dendritic cell-specific promoter activity score (DCSPA score)—is calculated as the difference between the observed RPU in DC2.4 cells and the sum of the observed relative promoter activities in all four off-target cells (Figure 4A).
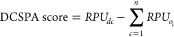
4where *RPU*_*dc*_ is the RPU in DC2.4 cells, and *RPU*_*o*_*c*__ is the RPU in an off-target cell, *c* is a serial identifier of off-target cells, and *n* is the number of off-target cells.

All 42 promoters exhibited a wide range of DCSPA scores ranging from ∼50 (1/39) to ∼−130 (1/17) (Figure 4A). A negative DCSPA score indicates the utilization of a transcriptional landscape not unique to DCs, while a positive DCSPA score indicates what portion of the observed RPU in the target cell is a result of the utilization of a DC-specific transcriptional landscape. Hence, we categorized the first library of promoters based on DCSPA scores as promoters exhibiting positive DCSPA scores (24) and promoters exhibiting negative DCSPA scores (18) (Figure 4A). As expected, all 11 DC-specific first library promoters exhibit a range of positive DCSPA scores (Figures 3A and 4A). Furthermore, promoter 1/42, which exhibited the highest RPU across all 42 promoters, has the second-lowest positive DCSPA score (Figures 3A and 4A). In general, no relationship could be established between the DCSPA score and the observed relative promoter activities in the target DC2.4 cells across all 42 promoters or all 24 promoters exhibiting positive DCSPA scores (data not shown).

Next, we proceeded to delineate how individual constituent TFREs influence DC-specific activity in a heterotypic synthetic promoter architecture. We calculated the DC-specific TFRE activity (DCSTA) score of each TFRE as the sum of the product of the DCSPA score of each promoter and the copy number of each TFRE within each promoter across all 42 promoters.
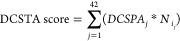
5where *N*_*i*_*j*__ is the copy number of a given TFRE *i* in a given promoter *j*, *DCSPA*_*j*_ is the DCSPA score of a given promoter *j*.

A ranking of all 12 TFREs in decreasing order of the DCSTA score indicated that, on the basis of TFRE composition, IRF9-A, IRF4-B, and BATF-B are the key contributors to the DC-specific activity, while BATF-A, JUNB-A, and IRF8-A elicit the most pronounced negative effects on DC-specific activities (Supporting Information, Figure S7). Other TFREs such as IRF4-A, c-Rel-B, c-Rel-A, RELA-A, STAT5A-A, and SPI1-A appear to exert the least effects on DC-specific activities (Figure 4B, Supporting Information, Figure S7). Using the normalized DCTSA score of each TFRE (*i.e.,* normalized by the DCTSA score of IRF9-A), we theoretically estimated the resultant contribution of constituent TFREs to DCSPA, that is, the estimated DCSPA score (Figure 4B,C). The estimated DCSPA score of each promoter is computed as the sum of the product of the normalized DCTSA score of each TFRE and the copy number of each TFRE across all TFREs within a given promoter.
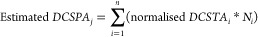
6where *n* is the number of TFREs within a given promoter *j*, and *N*_*i*_ is the copy number of a given TFRE within a given promoter *j*, *i* is a serial identifier of TFREs within a given promoter *j*.

A comparison of the estimated DCSPA score to the observed DCSPA score revealed a significant relationship between both values (*r*^2^ = 0.6806, *p* value < 0.0001). This strongly indicated that the observed DCSPA is primarily influenced by the TFRE composition and distinct transcriptional behaviors of constituent TFREs in a heterotypic environment (Figure 4C). Other secondary underlying factors influencing DCSPA are the degree of concentration of individual TFREs and cooperative transcriptional behavior of portions of corepresented TFREs within the promoter architecture.

First, promoters such as 1/02, 1/03, and 1/04, which are highly concentrated with a high DCSTA TFRE, IRF4-B (14, 10, 12 copies, respectively), have very low DCSPA scores owing to very low activities in the target DC2.4 cells (Figures 3A and 4A, Supporting Information, Table S10). Also, promoter 1/01, which is highly concentrated with SPI1-A motifs (10 copies), has a negative DCSPA score and has the lowest activity (∼1%) of all 42 first library promoters (Figures 3A and 4A, Supporting Information, Table S10). In fact, the activities of the aforementioned promoters are significantly lower than the individual activities of homotypic constructs of IRF4-B and SPI1-A in the target DC2.4 cells (Figures 2B and 3A). We speculate that excessive concentration of IRF4-B and/or SPI1-A within these promoters may have resulted in clustering or sequestration of the cognate TF(s), which is detrimental to TAs in the target DC2.4 cell. Conversely, promoter 1/41, which is highly concentrated with IRF9-A (12 copies) and IRF4-A (8 copies), has a high DCSPA score and high activity in DC2.4 cells (Figures 3A and 4A, Supporting Information, Table S10). This clearly demonstrates that the TFRE concentration range is a limiting factor that influences DCSPA. Furthermore, the observed pattern of promoter activities at a high concentration of different TFREs further indicates unique transcriptional behaviors of TFREs in a heterotypic promoter architecture.

Second, we hypothesize that some constituent TFREs could also positively influence DCSPA cooperatively where corepresented within the same heterotypic promoter architecture. To test this, we measured the differential frequency of representation of individual TFREs across promoters displaying positive and negative DCSPA scores in order to further delineate how constituent TFREs influence DCSPA. This is calculated as the difference between the total copies of each individual TFRE across promoters in each DCSPA category.
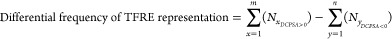
7where *m* and *n* are the number of promoters where a given TFRE occurs across promoters displaying positive and negative scores, respectively; *N*_*x*_*DCPSA*>0__ and *N*_*y*_*DCPSA*<0__ are the copy numbers of a given TFRE within a given promoter *x* displaying a positive DCSPA score and promoter *y* displaying a negative DCSPA score, respectively; *x* and *y* are serial identifiers of promoters displaying positive DCSPA and negative DCSPA scores, respectively.

**Figure 5:**
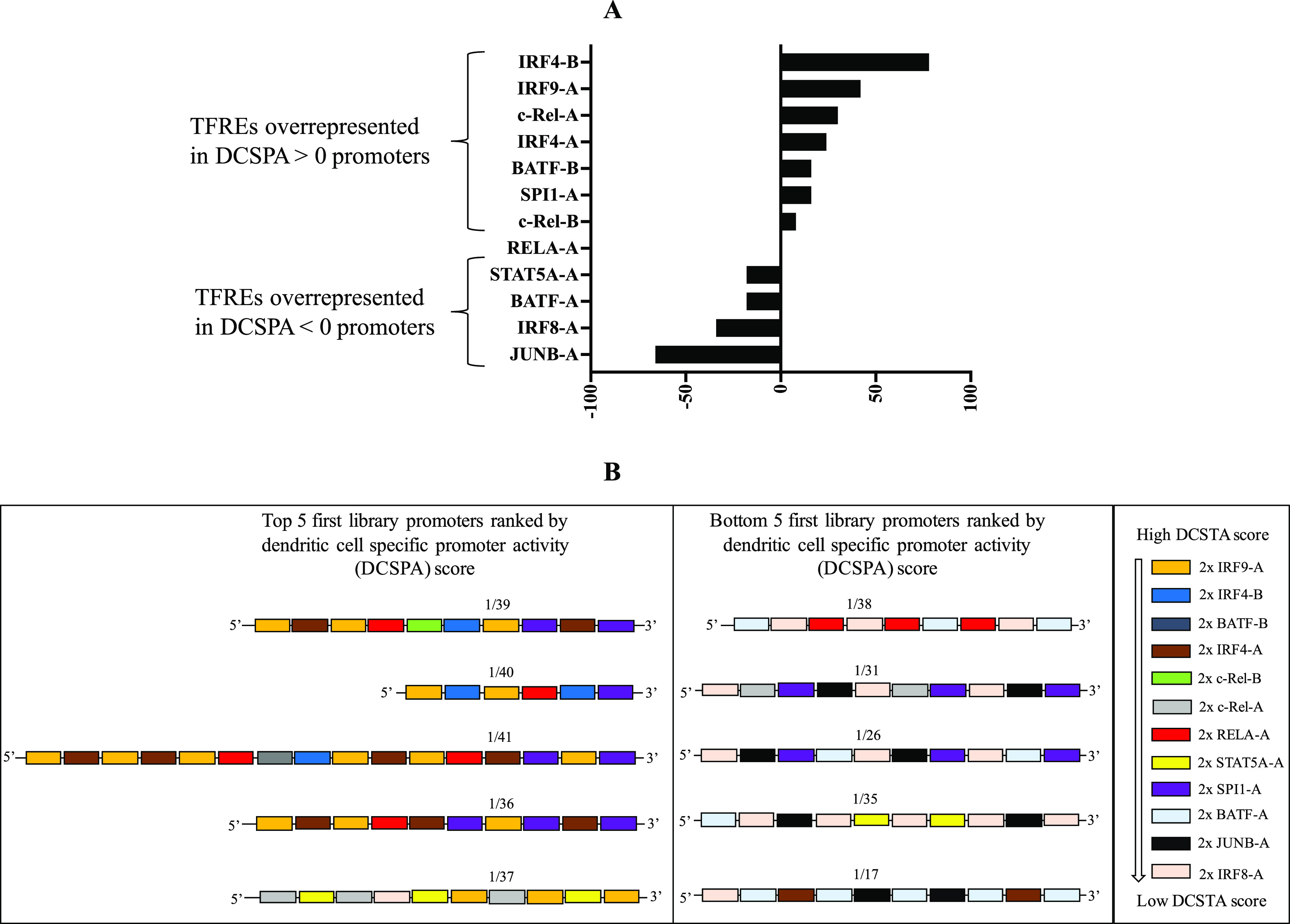
(A) Differential frequency of representation of individual TFREs across first library promoters indicates that IRF4-B, IRF9-A, c-Rel-A, IRF4-A, BATF-B, SPI1-A, and c-Rel-B are relatively overrepresented across first library promoters displaying positive DCSPA scores, while STAT5A-A, BATF-A, JUNB-A, and IRF8-A are relatively overrepresented across first library promoters displaying negative DCSPA scores. (B) Schematic illustration of the architecture of the top five and bottom five first library promoters ranked by DCSPA score showing a differential representation of TFREs across both promoter categories.

The differential frequency of the representation metric indicates that IRF4-B, IRF9-A, c-Rel-A, IRF4-A, SPI1-A, BATF-B, and c-Rel-B (*i.e.,* TFREs exhibiting positive differential frequency of representation), are relatively more represented across promoters exhibiting positive DCSPA scores compared to promoters exhibiting negative DCSPA scores (Figure 5A). However, while SPI1-A (PU.1 cognate TF) has a negative DCSTA score, its overrepresentation across promoters exhibiting positive DCSPA scores suggests that it potentially does impart positively on DCSPA *via* cooperation with some corepresented TFREs (Figures 4A and 5A).^42^ This further suggests that pairs or groups of TFREs which exhibit a positive differential frequency of representation may collectively influence positive DCSPA *via* cooperative interaction of cognate TFs on these TFREs, where corepresented within the same promoter architecture.

Conversely, the low DCSTA TFREs, STAT5A-A, BATF-A, IRF8-A, and JUNB-A, are relatively overrepresented across promoters exhibiting negative DCSPA scores, while RELA-A is equally represented across both promoter categories (Figures 4A and 5A). Following the same logic, STAT5A-A, BATF-A, IRF8-A, and JUNB-A may negatively impart the DC promoter activity, both cooperatively and individually. Furthermore, we speculate that the observed null differential frequency of representation of RELA-A suggests that it could cooperatively influence both DC-specific and non-DCSPA depending on the differential frequency of representation of corepresented TFREs within the promoter architecture (Figure 5A). Figure 5B depicts a differential representation of high and low DCSTA TFREs across the top five promoters with high DCSPA and the bottom five promoters with the lowest DCSPA, respectively.

### Constructing DC-Specific Promoters Using Design Rules Established from the First Library

Having delineated the unique transcriptional behavior of individual TFREs in a heterotypic environment and the underpinning role of the TFRE composition in DCSPA, we proceeded to generate a second library of DC-specific synthetic promoters exhibiting varied transcriptional activities. A number of design goals were set, including (i) generating a library of exclusively DC-specific promoters, which are architecturally distinct from the first library of promoters, (ii) utilizing optimal concentrations of individual TFREs required for the optimal target promoter activity and minimal off-target activities per promoter assembly, (iii) encoding high DC-specific transcriptional activities within minimal stretches of DNA, that is, minimal length. To achieve these objectives, promoters were designed predominantly with TFREs overrepresented across first library promoters exhibiting positive DCSPA scores. We also utilized single unpaired TFRE units in addition to 2-mer TFRE units as constituent building blocks from homotypic constructs in order to increase variability in the TFRE copy number. We aimed to attain optimal concentrations of TFRE blocks by restricting the maximum copy number of any given TFRE to six copies in order to avoid previously observed detrimental effects of high TFRE concentrations on the promoter activity. Adhering strictly to these design rules, we generated a second library of 16 promoters ranging from 119 to 434 bp in length (Supporting Information, Table S11).

**Figure 6:**
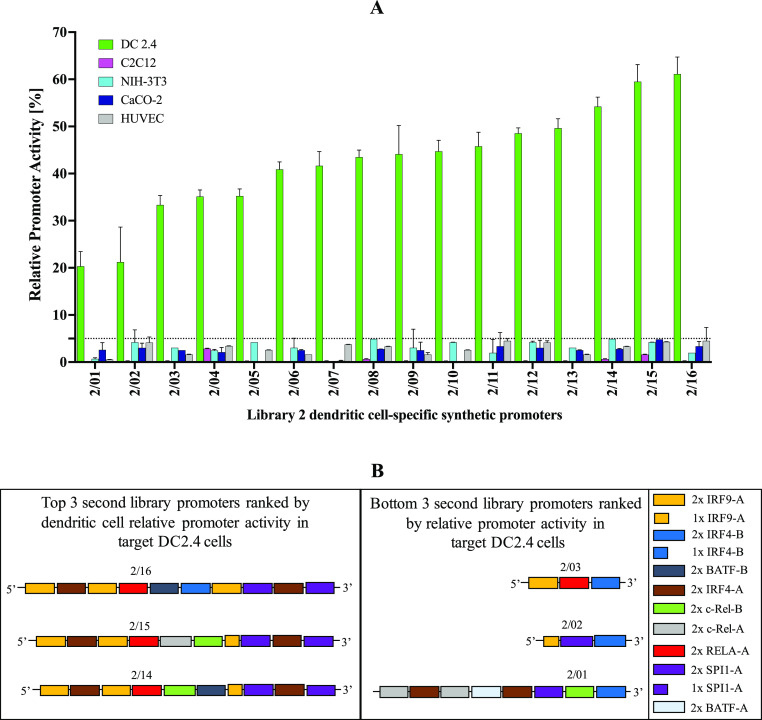
*In vitro* screening of a second library of exclusively DC-specific promoters. Promoter constructs are screened for GFP expression relative to the hCMV-IE1 promoter (positive control). The observed RPU of all 16 second library promoters in the target DC2.4 cells and all four off-target cells (C2C12, NIH-3T3, CaCO-2, HUVEC). Promoters display activities ranging ∼20–61% in the target DC2.4 cells and <5% in all off-target cells. Values represent the mean ± standard deviation across three independent transfections, each performed in triplicate. (B) Schematic illustration of the architecture of the top three and bottom three second library promoters ranked RPU in the target DC2.4 cells, showing the differential representation of TFREs across both promoter categories.

Upon screening this second library, all 16 promoters displayed relative promoter activities ranging from ∼20–∼61% in DC2.4 cells and <5% in all off-target cells relative to the activity of the hCMV-IE1 promoter (Figure 6A, Supporting Information Figure S8). Essentially, all 16 promoters display over 5-fold greater activity in the target DC2.4 cells relative to all off-target cells. Figure 6B illustrates the differential representation of TFREs across the strongest and weakest second library promoters.

### DC-Specific Synthetic Promoters Elicit a Range of Transcriptional Activities for User-Defined Functionality

**Figure 7:**
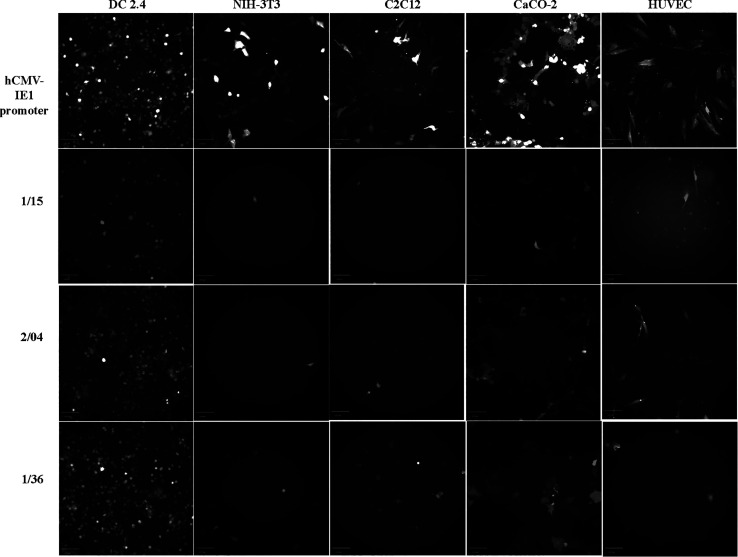
Fluorescence imaging of GFP expression in the live target (DC2.4) and off-target cells (NIH-3T3, C2C12, CaCO-2, and HUVEC) under the control of weak (1/15), moderate (2/04), and strong (1/36) DC-specific synthetic promoters relative to the hCMV-IE1 promoter, using the FITC channel setting on an INCELL Analyzer 2000. Image scale—100 μm.

Overall, we have generated 27 DC-specific synthetic promoters from 2 separate libraries of promoter assemblies (11 of 42 from the first library and 16 of 16 from the second library). A strong positive relationship is established between the observed relative promoter activities and the DCSPA scores of all 27 promoters (*r*^2^ = 0.9568, *p* value < 0.0001) (Supporting Information, Figure S9). This demonstrates that we can precisely tune the promoter strength in the target DC without compromising the cell-type specificity of the actuated TA. All 27 DC-specific synthetic promoters exhibiting over an 8-fold range of TA in the target DC2.4 cell line can be categorized as weak (5–29%), moderate (30–49%), and strong (>50%) promoters (Table 2). Figure 7 depicts a gradient of GFP expression in the target DC2.4 cells under the control of three representative promoters 1/15, 2/04, and 1/36 selected from both the promoter libraries. A minimal GFP expression is also shown across all four off-target cells under the control of all three representative promoters compared to a high GFP expression under the control of the pan-active hCMV-IE1 promoter (Figure 7). All DC2.4-specific synthetic promoters described here (119–600 bp) are much shorter in length when compared to the endogenous promoters of highly expressed DC-specific genes, such as the fascin gene promoter (3.5 kb),^17^ destin-2 promoter (3.2 kb),^13^ and CD11c, DC-SIGN, DC-STAMP, Langerin promoters (3–5 kb) (Table 2).^15^ Where recombinant DNA load capacity is a critical constraint (*e.g.,* in AAV vectors for gene therapy), synthetic promoters designed as described, therefore, offer a significant advantage in terms of transcriptional targeting and efficiency (TA per DNA length) (Table 2). Moreover, we show that it is possible to control TA while maintaining specificity.

**Table 2:** Description of All DC-specific Promoters and Categorization According to Promoter Strength

synthetic promoter	RPU in DC 2.4 cells [%]	strength category	size [bp]	TA per DNA length [%/bp]
1/06	8.39	weak	374	0.02
1/07	9.56		400	0.02
1/11	15.24		303	0.05
1/12	15.34		318	0.05
1/15	16.00		177	0.09
1/16	17.01		600	0.03
1/19	17.95		449	0.04
2/01	20.34		295	0.07
2/02	21.22		119	0.18
1/23	24.30		451	0.05
2/03	33.33	moderate	130	0.26
2/04	35.12		130	0.27
2/05	35.24		126	0.28
2/06	40.89		240	0.17
2/07	41.65		337	0.12
2/08	43.49		296	0.15
2/09	44.12		270	0.16
2/10	44.72		434	0.10
2/11	45.77		280	0.16
2/12	48.54		425	0.11
2/13	49.63	strong	367	0.14
1/36	50.00		471	0.11
2/14	54.21		409	0.13
2/15	59.50		405	0.15
2/16	61.11		356	0.17
1/39	66.37		454	0.15
1/40	66.70		274	0.24

## Conclusions

We have described a bottom-up approach to construct cell type-specific synthetic promoters displaying predictable activity and specificity solely by mining the transcriptional landscape of target and off-target cells. A combination of *in silico* and *in vitro* preliminary screening of genome-mined TFREs in both homotypic and heterotypic architectures enabled rapid identification of functionally active DNA motif sequences and prompt definition of an optimal design space for promoter assembly. We have essentially generated compact synthetic mimicries of endogenous DNA motif stretches which encode DC-specific gene expression without *a priori* empirical knowledge of transcriptional activities of individual constituent TFREs in an endogenous promoter environment. In particular, our approach obviates the need for intensive *in vitro* screening of large libraries of candidate promoter sequences, which is a common requirement in designing synthetic promoters with user-defined functionalities.^24−26^ More importantly, we delineate how TFRE composition primarily underpins activity and specificity of DC-synthetic promoters.

With over 300 TFREs (∼40 per 8 final TF candidates) mined from the endogenous promoters of candidate genes from our bioinformatics pipeline, we anticipate that the *in silico* promoter assembly process demonstrated here could generate a larger array of functionally relevant DC-specific synthetic promoters. This is immediately achievable by adopting DC-specific synthetic promoters reported here as design archetypes. Additionally, both our informatics workflow and *in silico* promoter assembly process may be adopted for the *de novo* design of synthetic promoters with transcriptional activities specific to any given cell type of interest.

## Materials and Methods

### Molecular Cloning for GFP Expression Reporter Vector Construction

The pVAX1 vector (ThermoFisher, UK), which contains a cytomegalovirus (CMV) promoter sequence, was used as the parental expression vector for constructing a reporter vector for promoter strength assays. Q5 Site-directed Mutagenesis Kit (New England Biolabs, UK) was used to insert *Nhe*I, *Kpn*I, *Mlu*I, *Bsu36*I, and *Nru*I unique restriction sites within the vector in order to facilitate restriction digestion–ligation cloning. The coding sequence (CDS) of a GFP from pmax-GFP (Lonza, Switzerland) was cloned into the vector between *Nhe*I and *Kpn*I restriction sites. The wild-type core promoter of the human cytomegalovirus immediate-early 1 gene (hCMV-IE1) was synthesized as a double-stranded DNA oligonucleotide (Eurofins) and inserted between *Bsu36*I and *Nhe*I upstream of the GFP CDS, thus replacing the original core element in pVAX1. The final expression vector—pVAX1-CMV-GFP—was used as the positive control vector for assaying the promoter strength (Supporting Information, Figure S3). Homotypic and heterotypic promoter sequences were synthesized as double-stranded dsDNA oligonucleotides (Genewiz, Germany) and inserted into pVAX1-CMV-GFP, between *Mlu*I and *Bsu36*I sites, thus replacing the hCMV-IE1 promoter upstream of the core promoter to create both homotypic and heterotypic expression vectors, respectively. Endotoxin-free, transfection-grade plasmid DNA is purified using the GenElute Endotoxin-free plasmid Midiprep kit (Merck, UK).

### Cell Culture and Transfection

DC2.4—murine monocyte-derived DC line (Merck Millipore, USA), NIH-3T3 (ECACC 93061524)—mouse embryonic fibroblast cell line, C2C12 (ECACC 91031101)—mouse muscle myoblast cell line, and CaCO-2 (ECACC 86010202)—human colon epithelial cell line (Public Health England, UK), and HUVEC—Human Umbilical Vein Endothelial Cells (Merck, UK) were used in this work. All cells were routinely cultured at 37 °C in 5% (v/v) CO_2_ in tissue culture-treated flasks in a humidified static incubator, according to the supplier’s instructions. DC2.4 cells were cultured in RPMI medium (Sigma) supplemented with glutamine (2 mM), fetal bovine serum (10%), herpes buffer solution (1×), nonessential amino acids (1×), and β-mercaptoethanol (0.0054×), and routinely subcultured at ∼85% confluency by seeding fresh media at 20,000 cells/cm^2^. NIH-3T3 cells were cultured in Dulbecco’s modified Eagle medium (DMEM) (Sigma), supplemented with glutamine (2 mM) and calf serum (10%), and subcultured when cells were ∼80% confluent by seeding fresh media at 30,000 cells/cm^2^. C2Cl2 cells were cultured in DMEM supplemented with glutamine (2 mM) and fetal bovine serum (10%) and subcultured when cells were ∼50% confluent by seeding fresh media at 2000 cells/cm^2^. CaCO-2 cells were cultured in Eagle’s minimum essential medium (Sigma, UK) supplemented with glutamine (2 mM), fetal bovine serum (10%), and nonessential amino acids (1×) and routinely subcultured when cells are ∼80% confluent by seeding fresh media at 30,000 cells/cm^2^. HUVEC cells were cultured in an endothelial cell growth medium (Sigma, UK) and routinely subcultured when cells are ∼80% confluent by seeding fresh media at 5000 cells/cm^2^. Cell viability and concentration were measured using a VI-CELL viability analyzer (Beckman-Coulter, USA) based on the trypan blue exclusion assay. Accutase (Merck, UK) was used to detach all adherent cells prior to subculturing or transfection.

All cells were transfected in a 96-well Amaxa Nucleofector System (Lonza, Switzerland) at ∼80% confluence, following the manufacturer’s protocol and instructions. Appropriate buffers and supplements were applied for each cell type as follows: DC2.4 and NIH-3T3 (SG buffer, supplement 1), C2C12 and CaCO-2 (SE buffer, supplement 1), and HUVEC (P5 buffer, supplement 3). Transfected cells were plated in black, flat-bottomed 96-well microplates (Greiner Bi-One, UK) after transfection and incubated at 37 °C, 5% CO_2_ for 24 h.

### *In Vitro* GFP Expression Measurement and Visualization

The GFP fluorescence measurement (*E*_x_ 485 nm, *E*_m_ 535 nm; bottom read) was performed using a SpectraMax iD5 microplate reader (Molecular Devices, UK). The relative fluorescence unit (RFU) was calculated by normalizing observed GFP fluorescence values with the fluorescence values from cell cultures transfected with pVAX1, that is, a GFP-null vector negative control. The RPU unit of all synthetic promoters in each cell type was calculated as a percentage of RFU values of pVAX1-CMV-GFP (positive control) in each cell type. GFP fluorescence imaging of live cells was performed with an INCELL Analyzer 2000 (GE Healthcare, USA) using channel settings for FITC. Transfection efficiencies were measured with a Countess 3 Automated Cell Counter (ThermoFisher Scientific, UK), following the manufacturer’s instructions.

## Supporting Information Available

Detailed bioinformatic analysis of gene expression data of target and off-target cells, TFRE composition and copy number of all synthetic promoters, vector map of the pVAX1-CMV-GFP plasmid, GFP expression fluorescence read-out, and DNA sequences of DC-specific synthetic promoters (PDF)
