# Supplementary material for: Bioinformatic Design of Dendritic Cell-Specific Synthetic Promoters
Source: ACS Synth Biol. 2022 Apr 7;11(4):1613–26. doi: 10.1021/acssynbio.2c00027 (PMC9016764; doi:10.1021/acssynbio.2c00027)
Supplement: Supplementary file 1 — sb2c00027_si_001.pdf [file sb2c00027_si_001.pdf]

SUPPLEMENTARY INFORMATION

Bioinformatic Design of Dendritic Cell-Specific Synthetic Promoters

Abayomi O. Johnson[1,2], Susan B. Fowler[3], Carl I. Webster[4], Adam J. Brown[1,2,*], David C. James[1,2,*]

[1]Department of Chemical and Biological Engineering, University of Sheffield, Mappin St., Sheffield, S1 3JD, United Kingdom
[2]SynGenSys Limited, Freeths LLP, Norfolk Street, Sheffield, S1 2JE, United Kingdom
[3]Antibody Discovery and Protein Engineering, R&D, AstraZeneca, Cambridge, CB21 6GH, United Kingdom
[4]Discovery Sciences, R&D, AstraZeneca, Cambridge, CB21 6GH, United Kingdom

*****Address correspondence to:

Professor David C. James:
Email, d.c.james@sheffield.ac.uk
Tel: +441142227594

Or

Dr. Adam J. Brown:
Email, adam.brown@sheffield.ac.uk
Tel: +441142227505

Table S1: Highly expressed genes in monocyte-derived dendritic cells; **Table S2:** Highly expressed genes in conventional dendritic cells; **Table S3:** Tissue cell type specificity settings on GeneVestigator V3; **Table S4**: Genes exhibiting high transcriptional activity and specificity in monocyte-derived dendritic cells and conventional dendritic cells; **Table S5:** Genes upregulated during activation/maturation of dendritic cells; **Table S6:** Categorisation of relevant dendritic cell genes; **Table S7:** Tissue specificity and detailed matrix description filter settings on MatInspector for APC specific TFREs; **Table S8:** Tissue specificity and detailed matrix description filter settings on MatInspector for non-APC specific TFREs; **Table S9:** Off-target cells types of off-target tissues for informatics and *in vitro* assay; **Figure S1:** Analysis of candidate dendritic cell-specific transcriptional factor response elements (TFREs) and transcriptional factors in monocyte-derived and myeloid dendritic cells; **Figure S2:** Identification of dendritic cell-specific transcriptional factors; **Figure S3:** Plasmid map of pVAX1-CMV-GFP; **Figure S4:** GFP fluorescence intensities of all homotypic promoters and the hCMV-IE1 reference promoter (CMV) in DC 2.4, C2C12, NIH-3T3 and HUVEC cells; **Table S10:** TFRE composition and copy number of a first library of 42 synthetic promoters; **Figure S5:** GFP fluorescence intensities of all 42 first library promoters and the hCMV-IE1 reference promoter (CMV) in (A) DC 2.4, (B) C2C12 and NIH-3T3, (C) Caco-2 and (D) HUVEC cells; **Figure S6**: Specificity of dendritic cell-specific transcriptional activity across all 42 first library promoters visualised as the minimum fold change between the relative promoter activity in DC2.4 and the relative promoter activity across all off-target cells. 11 promoters exhibiting over 5-fold changes (marked by dotted line) are identified as dendritic cell-specific promoters; **Figure S7:** The dendritic cell-specific TFRE activity (DCSTA) score of all 12 TFREs arranged in descending order of magnitude; **Table S11:** TFRE composition, copy number of a second library of 16 dendritic cell-specific synthetic promoters**; Figure S8:** GFP fluorescence intensities of all 16 second library promoters and the hCMV-IE1 reference promoter (CMV) in DC 2.4, C2C12, NIH-3T3 and HUVEC cells; **Figure S9:** A simple linear regression of dendritic cell specific promoter activity score and the observed relative promoter activities of all 27 dendritic cell-specific promoters showing a strong positive relationship between both variables.

Supplementary Tables

Table S1: A1 – Genes highly expressed in monocyte-derived dendritic cells (moDC), ArrayExpress database at EMBL-EBI (www.ebi.ac.uk/arrayexpress/experiments/E-MTAB-6192).

Gene	Mean mRNA level	STD
**SNORD13**	12.82909385	0.019833554
**FTL**	12.81008256	0.030539457
**HLA-DRA**	12.71935515	0.022687207
**CCL22**	12.64226067	0.05310759
**TYROBP**	12.538833	0.031129798
**FTH1P8**	12.5216531	0.070062705
**RAB7A**	12.33515827	0.016670746
**GRN**	12.29561328	0.026035347
**MALAT1**	12.22171597	0.038826023
**LYZ**	12.18757319	0.033674948
**PSAP**	12.16688414	0.002611445
**LAPTM5**	12.07567422	0.032336161
**SLC25A6**	11.99567117	0.026516769
**SRGN**	11.94129852	0.050420388
**TMSB10**	11.93426646	0.082738587
**A2M**	11.89327457	0.00888872
**ANXA2P2**	11.87314745	0.074617681
**OAZ1**	11.83120541	0.021382434
**TXN**	11.82877427	0.019515204
**ATP6V0C**	11.80895541	0.055630383
**MRC1**	11.79075018	0.086431474
**PLA2G7**	11.78402235	0.061951269
**SPP1**	11.70435942	0.066146666
**ATP6V0E1**	11.65368214	0.008684307
**ITGAX**	11.61468957	0.038858546
**FTH1P7**	11.56785087	0.046545406
**YBX1**	11.54218633	0.004389826
**PLXDC2**	11.49943535	0.042659063
**CSTB**	11.46819527	0.058868029
**HLA-B**	11.46657791	0.014442646
**CALR**	11.45100415	0.046847303
**ANPEP**	11.43859743	0.053405334
**RF01210**	11.43452348	0.081118907
**GM2A**	11.42517079	0.046897114
**FPR3**	11.41178038	0.0536685
**ALOX15**	11.39910845	0.04031236
**CD1A**	11.38504379	0.031473336

**GLIPR1**	11.35768803	0.053854828
**CAPNS1**	11.35647621	0.050903268
**LCP1**	11.34066594	0.053985991
**PPT1**	11.30519777	0.047041434
**HIST2H2AC**	11.29740247	0.039179784
**ATP6V1B2**	11.2846137	0.02416734
**IGSF6**	11.26713139	0.014139913
**TUBB**	11.25563173	0.046659269
**SLC7A11**	11.24953265	0.088179485
**CTSS**	11.20418681	0.060699726
**EEF2**	11.19994701	0.031060926
**CTSC**	11.18802129	0.106105711
**CSDE1**	11.17487798	0.004084117
**PRDX1**	11.16326323	0.024411849
**IQGAP1**	11.15854157	0.05037886
**PEA15**	11.15684002	0.061749477
**PARP4**	11.15539767	0.051879959
**CD74**	11.15536821	0.037297678
**ATP5F1B**	11.15371615	0.027546463
**MOB3A**	11.13417093	0.126391782
**AC092573.1**	11.11716193	0.033915874
**CST7**	11.10573724	0.145211491
**SAMHD1**	11.04819144	0.05727806
**HLA-DMB**	11.04459135	0.048702836
**HMOX1**	11.04298858	0.121070979
**NCF2**	10.99800199	0.035083902
**GPNMB**	10.9781378	0.052138523
**GPR183**	10.97190986	0.134262421
**ARL6IP5**	10.96683019	0.047351652
**RAB31**	10.95549893	0.078305513
**FABP4**	10.94988614	0.108421396
**CTSZ**	10.94683943	0.057501033
**CSF1R**	10.94017429	0.049116318
**DAD1**	10.93555656	0.075826779
**GMFG**	10.93311728	0.017811189
**FGL2**	10.9284917	0.050015122
**ASAP1**	10.92624379	0.041823118
**HSPA8**	10.92559414	0.066567253
**RHOA**	10.92491618	0.062192056
**PKM**	10.89754187	0.003455852
**RPS15**	10.8941833	0.028181445
**UBA52**	10.89254416	0.068400081

**CAP1**	10.87504025	0.040000286
**RPL4**	10.86158421	0.056148352
**HEXB**	10.85920535	0.070526851
**AC018738.1**	10.85772775	0.055465758
**CYP1B1**	10.85136126	0.048733784
**CD53**	10.83999796	0.035308423
**ITM2B**	10.83479686	0.054787878
**ACTG1**	10.82385507	0.009522531
**ACTN4**	10.81868858	0.03222306
**FCER1G**	10.81461242	0.067663483
**ACTR2**	10.7957423	0.058883459
**GPX4**	10.79299059	0.103733047
**ATP6AP1**	10.79243902	0.063185304
**CD1B**	10.78872106	0.109914564
**CXCL16**	10.76588835	0.07233349
**PFN1**	10.762334	0.034771034
**LITAF**	10.719734	0.078048179
**PLEK**	10.71668552	0.124052856
**SLC3A2**	10.71057889	0.05017224
**PTBP1**	10.69695775	0.018226285
**GNS**	10.69245304	0.025389602
**MFSD12**	10.59092363	0.167872234

Table S2: B1 – highly expressed genes in conventional dendritic cells (cDC).[1]

Gene	Mean TPM	STD
**MT-RNR2**	42966.774	14420.97035
**ACTB**	23602.422	2268.143758
**FTH1**	22312.567	2849.869842
**FTL**	17256.463	3322.378054
**CD74**	13926.964	5060.07214
**TMSB4X**	12839.941	1174.97525
**EEF1A1**	11998.758	1418.413392
**B2M**	9970.056	1841.150728
**OAZ1**	8892.439	4711.765554
**MT-CO2**	7822.693	1851.798165
**MT-CO1**	7242.711	964.5692788
**MT-ND1**	6676.426	1313.841627
**MALAT1**	5891.448	1642.452342
**COTL1**	5858.83	1526.976099
**HLA-DRA**	5045.631	1997.947524
**MT-ND4L**	4504.026	1048.344304
**RPL10**	4494.921	525.6296917
**PSAP**	4491.943	825.5429734
**TPT1**	4359.985	1093.530409
**IFI30**	3966.538	1334.489197
**CST3**	3486.39	914.834628
**HLA-B**	3434.084	1045.551338
**RPL8**	3408.282	819.0088507
**RPS19**	3398.998	884.0476649
**RPL13**	3356.337	362.5989013
**HLA-DPA1**	3283.981	855.2909276
**EEF1A1P5**	3265.927	1053.001141
**MT-ND2**	3209.876	314.1323442
**RPS27**	3115.577	242.3899326
**PABPC1**	3076.857	385.7277945
**RPL41**	3054.854	113.3969374
**DUSP1**	2897.001	1242.553889
**TMSB10**	2890.841	856.8382726
**MT-TS1**	2874.058	950.7887692
**MT-CYB**	2857.191	315.3013425
**LYZ**	2837.866	1915.668424
**FCGR3A**	2805.037	1380.026972
**MT-ND3**	2729.896	1692.883829
**ACTG1**	2710.907	351.17409
**RPL19**	2680.893	280.9368966

**RPL28**	2676.312	241.9280207
**UBC**	2604.189	1007.280844
**MT-RNR1**	2536.331	1090.527872
**RPL26**	2525.474	223.2530034
**CTSS**	2463.753	571.948714
**TYROBP**	2418.549	489.7737061
**VIM**	2375.781	422.0928965
**RPS18**	2311.133	628.5886066
**HLA-C**	2304.075	683.1588141
**SAT1**	2302.362	332.0210485
**MT-ND5**	2299.851	388.1322022
**RPS11**	2230.471	559.3492156
**RPL4**	2228.519	362.3731121
**MT-ND4**	2189.995	598.8416652
**ALDOA**	2178.22	514.4990156
**RPL18A**	2156.834	260.3224751
**PFN1**	2040.761	521.8521144
**NAPSB**	1989.478	1460.647847
**RPL3**	1985.305	333.2907581
**RPS16**	1972.806	483.7850603
**GAPDH**	1967.819	276.7296197
**RPS23**	1963.033	688.145049
**RPL13A**	1957.101	292.974744
**MT-CO3**	1941.779	488.0359058
**CFL1**	1937.166	331.4724289
**ITM2B**	1932.745	463.7466549
**FCER1G**	1853.647	490.5215902
**RPS6**	1839.882	341.7982697
**GNB2L1**	1772.021	260.0601655
**SERPINA1**	1731.143	412.223002
**RPLP1**	1715.837	363.6256727
**RPL15**	1662.473	160.9303156
**RPL31**	1644.151	427.5831962
**SH3BGRL3**	1634.711	247.9604116
**RPS12**	1628.5	219.0760056
**RPLP2**	1619.835	236.8882772
**RPS3A**	1619.324	322.7096218
**RPS8**	1615.35	220.8630282
**RPS24**	1578.088	532.8809178
**RPL27A**	1531.635	273.5495961
**HLA-DPB1**	1526.252	722.2324508
**RPL11**	1517.189	102.5195622

**PTMA**	1516.975	327.782518
**RPS15**	1499.445	306.4764055
**RPL37A**	1480	225.0701887
**RPL17**	1467.666	216.3186594
**IFITM2**	1462.532	391.6872604
**NACA**	1419.749	116.303063
**MT-ATP6**	1414.479	351.4040304
**S100A11**	1414.434	138.4340323
**RPS3**	1381.354	216.7214932
**H3F3B**	1349.338	396.7425552
**LAPTM5**	1324.891	315.5564936
**RPS4X**	1323.043	149.0322362
**RPL12**	1287.614	248.7468478
**RPS28**	1279.531	97.33175146
**RPL5**	1261.458	144.9402203
**CORO1A**	1249.319	243.250832
**RPL23A**	1242.916	214.4746579
**ARHGDIB**	1238.502	109.5347158
**EIF1**	1233.582	213.5154021
**RPL29**	1225.004	166.2150607
**LSP1**	1209.741	347.3706244
**JUNB**	1204.877	386.4596521
**RPS13**	1194.023	204.0626675
**NFKBIA**	1178.972	919.2822053
**NAP1L1**	1165.918	396.034932
**C10orf54**	1162.204	546.2711433
**S100A4**	1148.42	323.8930379
**RPL7A**	1138.249	186.4163829
**FOS**	1135.474	288.8276293
**CD52**	1124.742	302.481606
**RPS17**	1124.579	59.80189163
**RPS29**	1112.521	305.4494239
**ARPC2**	1106.482	194.3824432
**H3F3AP4**	1092.558	142.6616449
**MYL6**	1056.885	179.2535253
**TXNIP**	1052.215	305.9298604
**ITGB2**	1050.728	281.5828448
**HLA-A**	1046.889	214.517029
**S100A10**	1034.736	164.5005644
**RPL30**	1032.97	191.6999853
**RPS20**	1023.131	76.3241403
**RPL32**	1009.913	113.6199672

**RPL6**	1002.258	142.8519671
**EEF1G**	1001.328	84.77240158
**IFITM3**	995.563	591.6842787
**MT-ND6**	985.477	263.7080231
**YWHAZ**	970.697	298.7662475
**RPS14**	969.982	271.2514027
**UBA52**	968.321	185.2041913
**RPL23**	961.831	162.5918191
**RPS27A**	946.594	241.4911798
**RPL10A**	943.564	97.4258306
**RPS5**	911.814	137.748256
**HLA-E**	907.995	139.6551852
**RHOA**	902.827	197.538106
**RPS9**	900.699	189.4488818
**TSC22D3**	894.199	355.4597041
**RPL36A**	875.566	69.9132334
**ARPC1B**	872.763	178.1156057
**RPLP0**	866.41	186.1399269
**S100A6**	862.948	87.16276144
**RPL18**	854.224	80.74826286
**DDX5**	853.912	53.99353718
**RNASEK**	846.536	290.9586126
**RPL18AP3**	846.132	110.3611378
**PTPN6**	845.021	106.5266758
**RPL7**	842.577	186.4640208
**LST1**	840.022	174.2094246
**MT-ATP8**	838.662	131.5829353
**RPL41P5**	821.7465	441.2177727
**TPM3**	812.482	102.3043759
**ANXA2**	812.139	77.39971894
**RPL38**	798.632	168.1615573
**HLA-DRB1**	793.915	285.2917051
**ARPC3**	787.143	72.02070376
**RPL39**	786.506	236.4056909
**RPL9P9**	785.681	299.0953032
**RPS28P7**	781.408	162.5513959
**FAU**	771.415	116.8237796
**PGK1**	762.725	60.11768791
**RPL35**	759.586	101.4698555
**RPL34**	756.805	166.5449588
**FKBP1A**	753.772	135.1011024
**SLC25A6**	746.975	94.94690562

**WARS**	744.133	159.8199595
**MT-TY**	743.2775	800.044356
**RPS15A**	736.849	95.51933609
**EEF1B2**	730.996	68.88509576
**RPL14**	729.013	129.0851509
**RPS25**	728.099	240.1494377
**H3F3A**	726.656	118.7908356
**CALM2**	717.621	161.181487
**RPL36**	698.486	82.06505646
**FTLP3**	697.05	103.6036848
**RAB11A**	690.772	496.2841145
**TKT**	686.14	125.9283902
**CALM1**	681.81	98.33185394
**GRN**	679.764	177.9184477
**RPL37**	677.851	147.5866886
**RPS10**	671.389	40.07567208
**HNRNPA1**	662.514	115.9671781
**LCP1**	659.12	107.7616474
**AIF1**	658.334	156.0027193
**TUBA1B**	645.963	72.00037106
**GABARAP**	641.414	167.0134009
**UCP2**	640.251	140.7431129
**RAC2**	638.816	91.34319039
**TCIRG1**	632.557	198.1804249
**FCN1**	632.433	184.4935677
**PTMAP5**	626.776	583.7713374
**KLF6**	625.78	143.5839453
**ZFP36**	615.548	261.3214994
**SARAF**	615.04	151.2988339
**FGR**	613.805	140.3512444
**RPSA**	597.677	162.6314997
**RPL27**	596.794	105.5388368
**PPIA**	587.543	117.0939219
**UBB**	581.639	29.33642071
**SELPLG**	578.137	140.0763572
**NFKBIZ**	573.314	371.1932812
**CD37**	558.597	104.6241773
**LINC01272**	552.137	65.00657348
**LILRB2**	544.394	105.7245779
**COX4I1**	542.649	132.8564255
**PFDN5**	534.845	120.7706051
**EIF4A1**	534.734	143.3953175

**GSTK1**	534.546	288.6918469
**PLAC8**	532.769	363.0268478
**CSF1R**	523.616	198.5509512
**PRELID1**	523.101	126.4001887
**SYNGR2**	521.989	62.54570883
**PTPRC**	521.344	87.9197016
**ACTR3**	508.286	148.692854
**EEF1D**	501.968	129.8200873
**HCK**	500.046	61.10773636
**HLA-DQB1**	499.456	434.007286
**DAZAP2**	499.259	58.83250265
**RHOG**	483.404	106.8651462
**ATP5B**	482.594	34.12003946
**RPS2**	479.249	118.5444676
**TAGLN2**	477.225	148.8387927
**FXYD5**	475.876	63.72703129
**RPL9**	469.161	128.0937107
**CD68**	466.833	119.4811942
**EMP3**	463.214	184.5850876
**CAP1**	461.548	39.72004541
**CFD**	461.191	213.8682186
**HMGN2**	457.769	24.18084376
**RPS21**	451.567	43.08443594
**SRGN**	448.28	167.7409811
**LRRC25**	447.002	96.04187736
**FLNA**	446.386	153.8714904
**CFP**	445.869	38.78773298
**STXBP2**	443.39	164.6625883
**FTH1P7**	438.356	90.18284554
**TSPO**	437.186	52.29109178
**HNRNPK**	434.761	104.0166374
**RPL41P1**	428.709	377.6223588
**EEF2**	418.112	49.11842992
**TPI1**	418.092	75.90407636
**LGALS1**	416.813	178.563663
**SERF2**	416.752	113.8818348
**CRIP1**	415.005	120.8967234
**HSPA8**	410.919	158.2102742
**RPS7**	408.006	87.23245521
**BTF3**	405.886	45.00052589
**ENO1**	401.115	71.18225104
**TUBA1A**	399.995	53.91499351

**SLC25A3**	399.738	63.82276598
**PECAM1**	395.844	158.2210938
**TIMP1**	394.45	200.3888347
**MS4A7**	392.724	98.09335843
**CDC37**	387.8	187.2846465
**YWHAB**	383.839	47.5748742
**PSME1**	383.576	65.80713652
**EIF3L**	376.906	116.2921104
**EEF1A1P6**	374.766	87.17357652
**RNH1**	372.957	30.84741796
**TNFRSF1B**	372.325	113.6697142
**S100A9**	370.817	249.8898452
**MTND2P28**	369.15	108.0809397
**RNASET2**	365.613	82.43609924
**RPL13AP5**	364.93	91.79719515
**RPL3P4**	360.173	345.8144113
**RPL35A**	359.274	117.8652023
**RPL21**	357.689	64.28564949
**MYD88**	356.41	40.75486336
**ANXA5**	354.614	50.88584201
**MIR3064**	352.805	410.4843965
**LILRA1**	351.764	77.53233796
**CD44**	349.701	53.64455289
**UQCRC1**	345.322	63.90300322
**GMFG**	343.255	209.6021637
**GSTP1**	340.212	76.0784713
**ADGRE5**	339.165	39.22627579
**HCLS1**	337.611	57.17858585
**ASAH1**	336.629	69.36020314
**SRSF5**	334.098	61.6364059
**CNBP**	332.278	35.11398628
**RHOC**	331.783	56.57244102
**MYL12A**	326.111	86.56905483
**MSN**	326.036	77.13287491
**CLIC1**	325.363	73.46900559
**CTSH**	321.403	102.6468551
**ARF1**	318.352	52.98826316
**CTSB**	318.019	52.78118964
**NPC2**	317.263	80.68432868
**RGS19**	312.725	78.69306299
**MT-TQ**	306.963	636.5700552
**CCNI**	300.48	108.5921367

**RPL24**	295.087	104.8358908
**PYCARD**	292.803	47.78565234
**MT-TN**	291.007	155.4957792
**CD48**	290.036	55.88341675
**TMBIM6**	288.69	61.35639952
**CTSD**	285.362	104.128723
**CPVL**	284.087	129.68021
**SIGLEC10**	282.607	101.8507773
**CD53**	281.285	77.61424966
**LIMD2**	277.98	79.70791115
**SLC25A5**	272.158	54.80868392
**ATP5G2**	272.019	73.2911407
**PGAM1**	270.935	22.76309486
**EIF3F**	268.597	46.01024894
**PTP4A2**	267.324	57.81571104
**GDI2**	265.049	64.69550317
**CCND3**	261.134	79.32295344
**BID**	260.933	31.67813769
**PILRA**	260.582	26.70503525
**MYL12B**	257.382	15.34261329
**SPN**	256.609	33.67900448
**ZFP36L1**	256.409	135.1624944
**PPP1CA**	254.65	13.2836756
**ATP6V0B**	253.126	23.01402871
**HLA-DQA1**	250.483	122.9385742
**SLC11A1**	248.485	65.07453294
**MYO1F**	247.207	60.34528757
**M6PR**	245.988	51.51361356
**NME2**	245.784	22.88393634
**CAPZB**	245.305	20.48321325
**ARPC5**	244.365	60.84653061
**HLA-F**	242.502	141.1998471
**LDHA**	242.316	44.77327264
**HLA-DRB5**	240.386	182.494776
**EIF5A**	240.231	42.63392478
**C1orf162**	237.759	36.4618188
**ANXA1**	237.203	93.36220124
**EIF3K**	236.913	52.59056683
**GPX4**	235.382	27.89436068
**MT-TL1**	232.734	152.4164751
**LILRB1**	232.662	67.81292932
**LILRA5**	231.535	84.35525658

**COX7C**	229.809	57.08853085
**PRR13**	229.577	40.61869068
**CTSC**	229.539	51.20306478
**PTMAP2**	228.379	83.78501159
**LY6E**	226.575	93.74953
**CMTM6**	226.129	35.72681038
**ATP5A1**	226.128	67.28538601
**AP1S2**	225.289	61.95950607
**CNN2**	224.757	43.19310747
**ATP5L**	224.755	58.27021774
**ZFAND5**	223.177	64.22947256
**RBM3**	220.725	42.33726063
**LGALS9**	217.434	76.71170131
**RNF130**	214.986	22.58902593
**RPS3AP6**	214.627	88.40512362
**MT-TK**	214.285	293.4270925
**PTGES3**	213.152	18.03307572
**THEMIS2**	213.035	51.41989875
**GNG5**	212.86	47.00170529
**ARPC4**	211.813	40.17305029
**CANX**	210.54	110.5558746
**IER2**	210.321	69.33293656
**NPM1**	210.021	61.52320887
**SLC44A2**	208.954	77.65790844
**PPT1**	207.154	48.64952433
**ID2**	206.266	32.60451146
**MT-TE**	205.557	200.0159337
**HNRNPDL**	205.209	38.58289945
**FCGRT**	204.861	35.16592801
**EIF3H**	204.307	38.3162982
**ZYX**	202.838	61.723497
**COX5A**	202.532	25.83490459
**RAP1B**	202.223	44.72667543
**IL16**	202.039	89.29424263
**AMPD2**	199.901	76.58821012
**CKB**	198.72	87.37614592
**LAMTOR4**	197.929	43.94854796
**LGALS3**	195.918	26.91009165
**ATG3**	195.773	43.29726227
**PPP1R15A**	194.7075	82.73655586
**NINJ1**	194.687	68.89701042
**MTATP6P1**	194.296	66.26750999

**ATP1B3**	193.132	11.41380338
**TALDO1**	192.716	28.85840311
**FCGR3B**	191.628	282.081323
**ITGA4**	191.303	53.97940922
**LYPD2**	190.083	73.48505814
**PSMB8**	189.751	39.44031478
**PKM**	189.082	40.01716516
**HMOX1**	188.489	38.23212318
**HK3**	187.794	19.59063022
**FAM26F**	186.825	101.5140354
**CD4**	186.756	25.51471791
**PPIAP22**	186.394	65.33823167
**FLII**	186.336	36.60852981
**ITGAX**	185.884	37.2570192
**LRRFIP1**	185.389	45.52638293
**GLIPR1**	183.732	28.31202077
**SHKBP1**	183.566	33.15337566
**TPP1**	183.307	42.34351081
**BIN2**	182.147	32.46568893
**GPX1**	181.902	63.5262592
**SLC7A7**	181.867	36.96713838
**PGLS**	181.61	38.89497365
**HNRNPA2B1**	178.462	29.0941458
**HLA-DMA**	178.214	88.53689664

Table S3: Tissue cell type specificity settings on GeneVestigator V3 [2]

Expressed in	Not expressed in
**Monocyte derived dendritic cells**	Blood outgrowth endothelium
	Blood vessel vascular endothelium
	Microvascular endothelium
	Lymphatic microvessel endothelium
	Skin fibroblast
	Dermal microvessel endothelium
	Epidermal keratinocyte
	Dermal fibroblast
	Monocyte derived microphage
	Skeletal muscle myoblast
	Skeletal muscle myotube
	Myoblast derived myotube
	Synovial fibroblast
**Conventional dendritic cells**	Blood vessel vascular endothelium
	Dermal microvessel endothelium
	Epidermal keratinocyte
	Dermal fibroblast
	Monocyte derived microphage
	Skeletal muscle myoblast
	Skeletal muscle myotube
	Myoblast derived myotube
	Joint synovial fibroblast

Table S4: Genes exhibiting high transcriptional activity and specificity in monocyte-derived dendritic cells and conventional dendritic cells [2]

Cell type	Gene
**Monocyte-derived dendritic cells**	CCL22
	ALOX15
	NCF2
	CD1A
	CD74
	TYROBP
	HLA-DRA
	CD1B
	LCP1
	FPR3
	MRC1
	CSF1R
**Conventional dendritic cells**	CPVL
	ITGB2
	AMPD2
	COTL1
	VSIR
	CD48
	FGR
	STXBP2
	MYO1F
	THEMIS2
	HCLS1
	CD74
	SPN
	TNFRSF1B
	FOS
	SELPLG
	SIGLEC10
	PLAC8
	HK3
	TCIRG1
	LRRC25
	RNASET2
	C1orf162
	CFP
	HLA-DRA
	FCN1
	RGS19
	PTPN6
	ADGRE5
	HLA-DPA1
	ITGAX
	HLA-DMA
	HLA-DPB1

Table S5: 72 C - Genes upregulated during activation/maturation of dendritic cells.[3]

Gene	Ensembl ID	Gene ID
**AZI2**	ENSG00000163512	64343
**B3GNT2**	ENSG00000170340	10678
**CCDC50**	ENSG00000152492	152137
**CCL2**	ENSG00000108691	6347
**CCL4**	ENSG00000129277	6351
**CD200**	ENSG00000091972	4345
**CD38**	ENSG00000004468	952
**CD80**	ENSG00000121594	941
**CD86**	ENSG00000114013	942
**CFB**	ENSG00000243649	629
**CFLAR**	ENSG00000003402	8837
**CLIC4**	ENSG00000169504	25932
**CXCL10**	ENSG00000169245	3627
**CXCL9**	ENSG00000138755	4283
**DNAJA2**	ENSG00000069345	10294
**DUSP2**	ENSG00000158050	1844
**EIF2AK2**	ENSG00000055332	5610
**FSCN1**	ENSG00000075618	6624
**GADD45B**	ENSG00000099860	4616
**GBP2**	ENSG00000162645	2634
**GCH1**	ENSG00000131979	2643
**GPD2**	ENSG00000115159	2820
**IFI44**	ENSG00000137965	10561
**IFIH1**	ENSG00000115267	64135
**IFIT2**	ENSG00000119922	3433
**IFIT3**	ENSG00000119917	3437
**IL15**	ENSG00000164136	3600
**IL15RA**	ENSG00000134470	3601
**IL2RA**	ENSG00000134460	3559
**IL6**	ENSG00000136244	3569
**IRF1**	ENSG00000125347	3659
**ISG20**	ENSG00000172183	3669
**JDP2**	ENSG00000140044	122953
**LTA**	ENSG00000226979	4049
**MARCKSL1**	ENSG00000175130	65108
**MX1**	ENSG00000157601	4599
**NCK2**	ENSG00000071051	8440
**NFKBIA**	ENSG00000100906	4792
**NMI**	ENSG00000123609	9111
**NT5C3**	ENSG00000122643	51251

**OAS2**	ENSG00000111335	4939
**OASL**	ENSG00000135114	8638
**PARP9**	ENSG00000138496	83666
**PELI1**	ENSG00000197329	57162
**PI4K2B**	ENSG00000038210	55300
**PLA1A**	ENSG00000144837	51365
**PML**	ENSG00000140464	5371
**PTGS2**	ENSG00000073756	5743
**RBBP8**	ENSG00000101773	5932
**REL**	ENSG00000162924	5966
**RGS1**	ENSG00000090104	5996
**RIPK2**	ENSG00000104312	8767
**RSAD2**	ENSG00000134321	91543
**RTP4**	ENSG00000136514	64108
**SDC4**	ENSG00000124145	6385
**SERPINB9**	ENSG00000170542	5272
**SOS1**	ENSG00000115904	6654
**STAT1**	ENSG00000115415	6772
**STAT2**	ENSG00000170581	6773
**TANK**	ENSG00000136560	10010
**TAP1**	ENSG00000168394	6890
**TDRD7**	ENSG00000196116	23424
**TLK2**	ENSG00000146872	11011
**TMCC3**	ENSG00000057704	57458
**TNFAIP3**	ENSG00000118503	7128
**TNFSF10**	ENSG00000121858	8743
**TNFSF4**	ENSG00000117586	7292
**TNFSF9**	ENSG00000125657	8744
**TRAF1**	ENSG00000056558	7185
**TRIM21**	ENSG00000132109	6737
**UBE2L6**	ENSG00000156587	9246
**XRN1**	ENSG00000114127	54464
**ZNF281**	ENSG00000162702	23528

Table S6: Categorisation of relevant dendritic cell genes

Gene Class Nomenclature	Gene Type	Number of genes
A1	Highly expressed in moDC	100
A2	Highly specific to moDC	100
A1-2	Highly expressed moDC-specific gene	12
B1	Highly expressed in cDC	400
B2	Highly specific to cDC	400
B1-2	Highly expressed cDC-specific gene	33
C	Upregulated in moDC & cDC	64

Table S7: Tissue specificity and detailed matrix description filter settings on MatInspector for APC specific TFREs

Contains	AND	Does not contain (ANY)
Antigen presenting cells		Skeletal muscles
		Skeletal
		Connective tissue
		Repressor
		Repressing
		Suppressor

Table S8: Tissue specificity and detailed matrix description filter settings on MatInspector for non-APC specific TFREs

Contains (ANY)	OR	Does not contain (ANY)
Skeletal muscles		Antigen presenting cells
Skeletal		Leukocytes
Connective tissue		PU.1
Repressor		
Repressing		
Suppressor		
Ubiquitous		

Table S9: Off-target cells types of off-target tissues for informatics and *in vitro* assay

Tissue	Cell types	Cell line for assay
Skeletal muscle tissue	Skeletal muscle	C2C12
	Skeletal muscle superior quadracep	
Connective tissue	Differentiated myofibroblast	NIH-3T3
	Skin cultured fibroblast	
	Skin fibroblast	
Endothelium	Microvascular endothelial cell	HUVEC
	Lymphatic vessel endothelial cell	
Epithelium	Skin epithelial cell keratinocyte	CaCO-2

Figure S1. Analysis of candidate dendritic cell-specific transcriptional factor response elements (TFREs) and transcriptional factors in monocyte-derived and myeloid dendritic cells (A) Identification of 745 transcriptional factor regulatory sequences (TFREs) over-represented in the promoter regions of target genes (highly expressed genes specific to the target cell types: monocyte derived dendritic cells (moDC), conventional/myeloid dendritic cells (cDC) and genes which are upregulated during dendritic cell activation/maturation), using the common TF tool of Genomatix, *p* value < 0.005. (B) Identification of 52 TFRES of transcriptional factors (TFs) unique to antigen presenting cells (APCs) in the promoter region of target genes, using the MatInspector tool of Genomatix. (C) Identification of 170 transcriptional factors highly expressed in the target cell types (moDC, cDC, and mature/activated myeloid/conventional dendritic cells (mmDC). (D) Selection of 79 candidate TFs which are relatively highly expressed in the target cell types compared to the off-target cell types (myocytes, fibroblast, endothelial and epithelial cells).

A

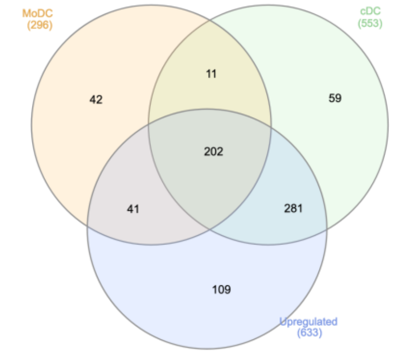

745 Common TFREs

B

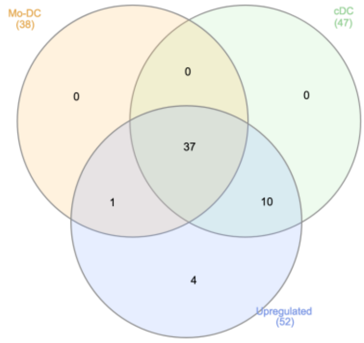

52 APC-related TFREs

C

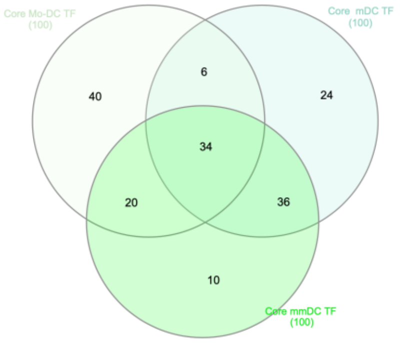

170 TFs across Mo-DC, myeloid DC, activated myeloid DC

D

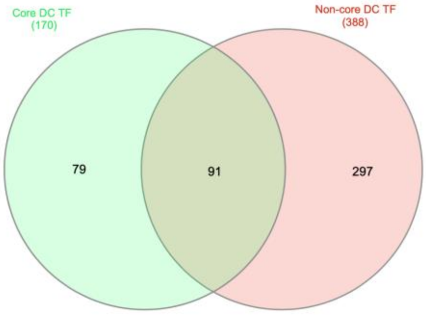

79 signature DC TF candidates

Figure S2. Identification of dendritic cell-specific transcriptional factors **(A)** Identification of 69 core transcriptional factors (TFs) with high regulatory potential in target cell types: monocyte-derived, conventional and activated dendritic cells (TSSQ cut-off > 0.1) using the Target Score Specificity Quotient (TSSQ) parameter *i.e.* the normalised difference of mean specificity score (1-1-100) of each of the 79 core TFs in the off-target and target cell types *i.e.* difference between the mean specificity score of each TF in all off-target cell types (MBSS) and the mean specificity score of each TF in all target cell types (MTSS) normalised the highest difference value. **(B)** Selection of 10 TFs with TSSQ > 0.1 and high regulatory potential in target cell types (using TFdiff encode (p value <0.05), Ingenuity Pathway Analysis, and the Human Protein Atlas) from a selection of transcriptional factor regulatory sequences (TFREs) which are common to a population of 745 over-represented TFREs in the promoters of target genes, 52 TFREs of TFs peculiar to antigen presenting cells (APCs), and all TFREs of the 69 core transcriptional factors found in the promoter region of target genes. **(C)** Classification of the 10 selected TFs based of TSSQ value **(D)** The mean of the mean specificity score of all 10 selected TFs in the off-target/base cell types and target cell types indicates that the selected TFs are unique to the target cell types (two-tailed independent t test, *p* value < 0.0001).

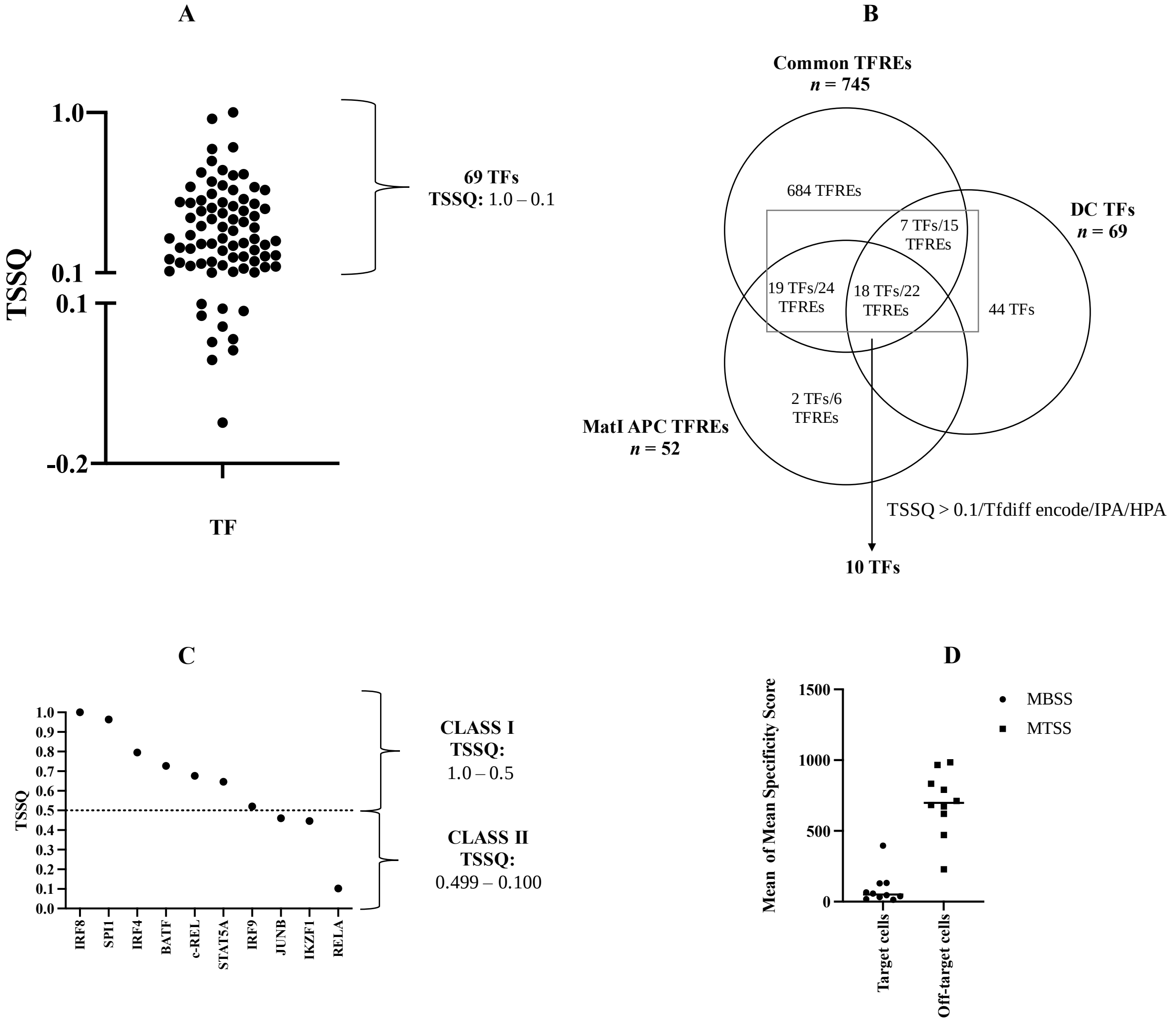

Figure S3: Plasmid map of pVAX1-CMV-GFP

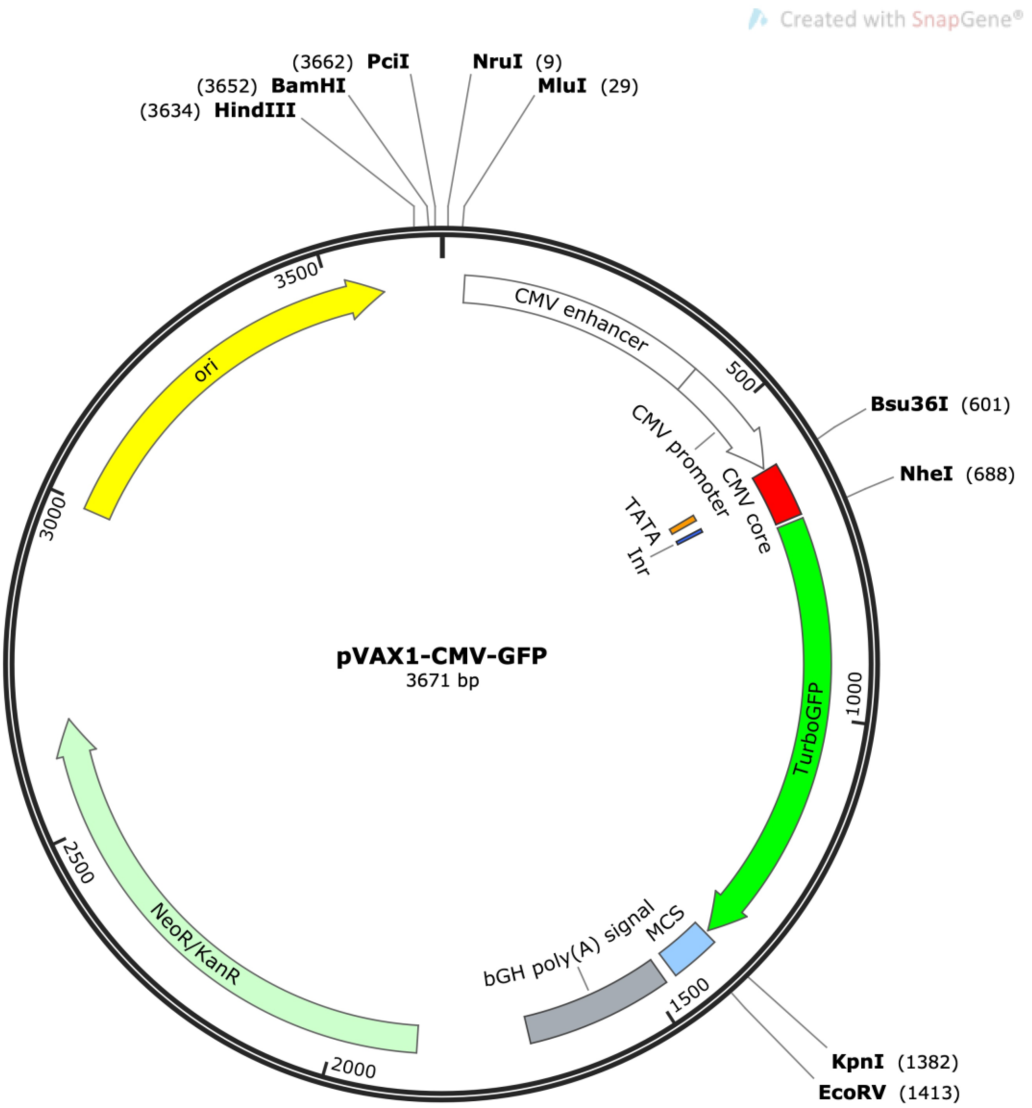

Figure S4: GFP fluorescence intensities of all homotypic promoters and the hCMV-IE1 reference promoter (CMV) in DC 2.4, C2C12, NIH-3T3 and HUVEC cells. Values represent the mean ± standard deviation across three independent transfections each performed in triplicate.

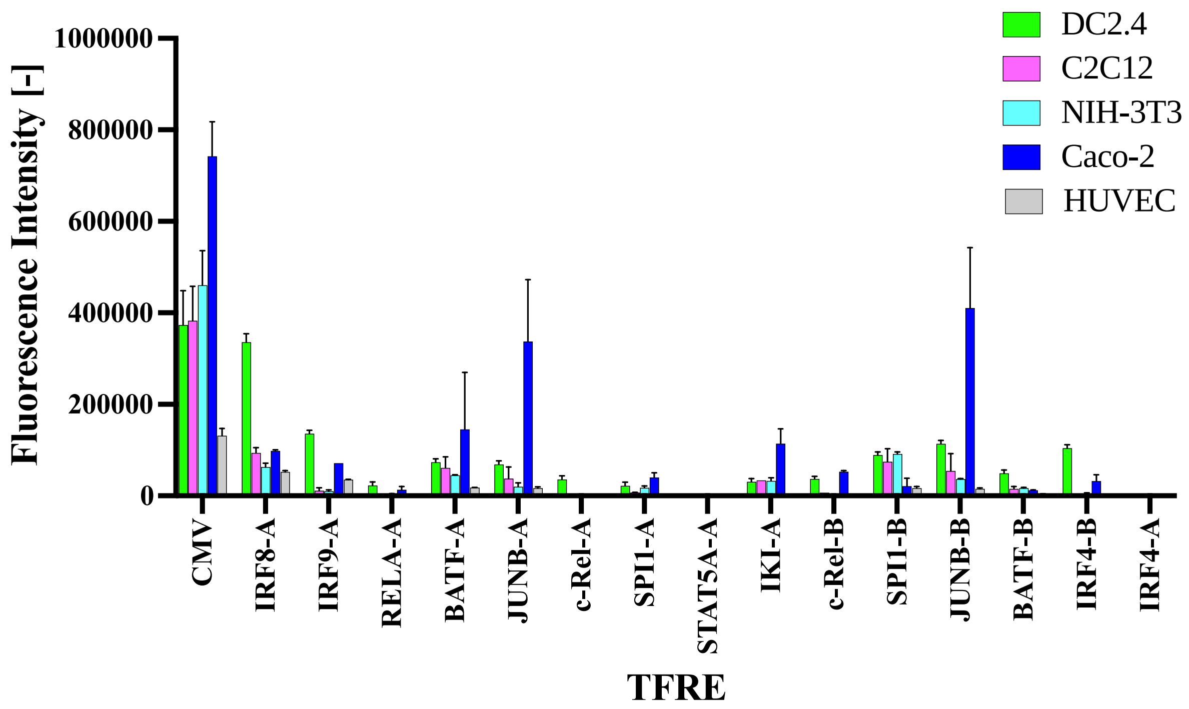

Table S10. TFRE composition and copy number of a first library of dendritic cell-specific synthetic promoter candidates

PROMOTER	IRF8-A	IRF9-A	RELA-A	BATF-A	JUNB-A	c-Rel-A	SPI1-A	STAT5A-A	c-Rel-B	BATF-B	IRF4-B	IRF4-A
**1/01**	0	0	0	2	0	0	10	0	0	0	0	4
**1/02**	0	0	2	0	0	2	2	0	2	2	14	2
**1/03**	0	0	0	0	0	4	4	0	0	2	10	0
**1/04**	0	6	2	0	0	6	6	0	0	6	12	0
**1/05**	0	0	0	0	6	0	0	6	0	0	0	6
**1/06**	0	0	0	0	0	6	6	0	0	0	6	0
**1/07**	0	0	0	0	0	6	6	0	0	2	6	0
**1/08**	0	0	0	2	0	6	6	0	0	0	0	6
**1/09**	2	0	0	0	4	0	10	0	0	0	0	4
**1/10**	0	0	6	0	4	0	6	4	0	0	0	0
**1/11**	0	0	0	0	0	0	2	0	2	2	6	2
**1/12**	0	0	0	0	0	6	0	0	0	6	6	2
**1/13**	0	4	0	0	6	0	4	0	0	0	0	6
**1/14**	0	6	0	0	0	0	6	0	0	2	6	0
**1/15**	0	0	0	0	0	2	2	0	0	0	2	2
**1/16**	0	6	2	0	0	8	2	0	2	2	8	0
**1/17**	4	0	0	10	4	0	0	0	0	0	0	4
**1/18**	2	0	0	0	4	10	0	0	0	0	0	4
**1/19**	0	4	0	0	0	2	4	0	0	2	6	2
**1/20**	0	6	2	0	0	0	6	0	0	0	6	0
**1/21**	2	0	0	0	4	10	0	4	0	0	0	0
**1/22**	0	0	0	0	6	4	4	6	0	0	0	0
**1/23**	0	4	2	0	0	0	4	0	0	0	6	2
**1/24**	2	0	0	0	6	6	6	0	0	0	0	0
**1/25**	0	2	0	0	0	6	0	0	0	6	6	0

**1/26**	6	0	0	4	4	0	6	0	0	0	0	0
**1/27**	0	10	0	2	0	0	0	4	0	0	0	4
**1/28**	0	6	0	0	0	6	6	0	0	0	0	0
**1/29**	0	10	0	2	4	0	0	0	0	0	0	4
**1/30**	6	4	0	0	6	0	6	0	0	0	0	0
**1/31**	6	0	0	0	4	4	6	0	0	0	0	0
**1/32**	0	6	0	2	0	0	6	0	0	0	0	6
**1/33**	2	6	0	0	6	6	0	0	0	0	0	0
**1/34**	0	10	2	0	4	0	0	4	0	0	0	0
**1/35**	10	0	0	2	4	0	0	4	0	0	0	0
**1/36**	0	6	2	0	0	0	6	0	0	0	0	6
**1/37**	2	6	0	0	0	6	0	6	0	0	0	0
**1/38**	6	0	6	6	0	0	0	0	0	0	0	0
**1/39**	0	6	2	0	0	0	4	0	2	2	2	4
**1/40**	0	4	2	0	0	0	2	0	0	0	4	0
**1/41**	0	12	2	0	0	2	4	0	0	0	2	8
**1/42**	8	0	0	2	2	0	2	0	0	0	0	2

Figure S5: GFP fluorescence intensities of all 42 first library promoters and the hCMV-IE1 reference promoter (CMV) in (A) DC 2.4, (B) C2C12 and NIH-3T3, (C) Caco-2 and (D) HUVEC cells. Values represent the mean ± standard deviation across three independent transfections each performed in triplicate.

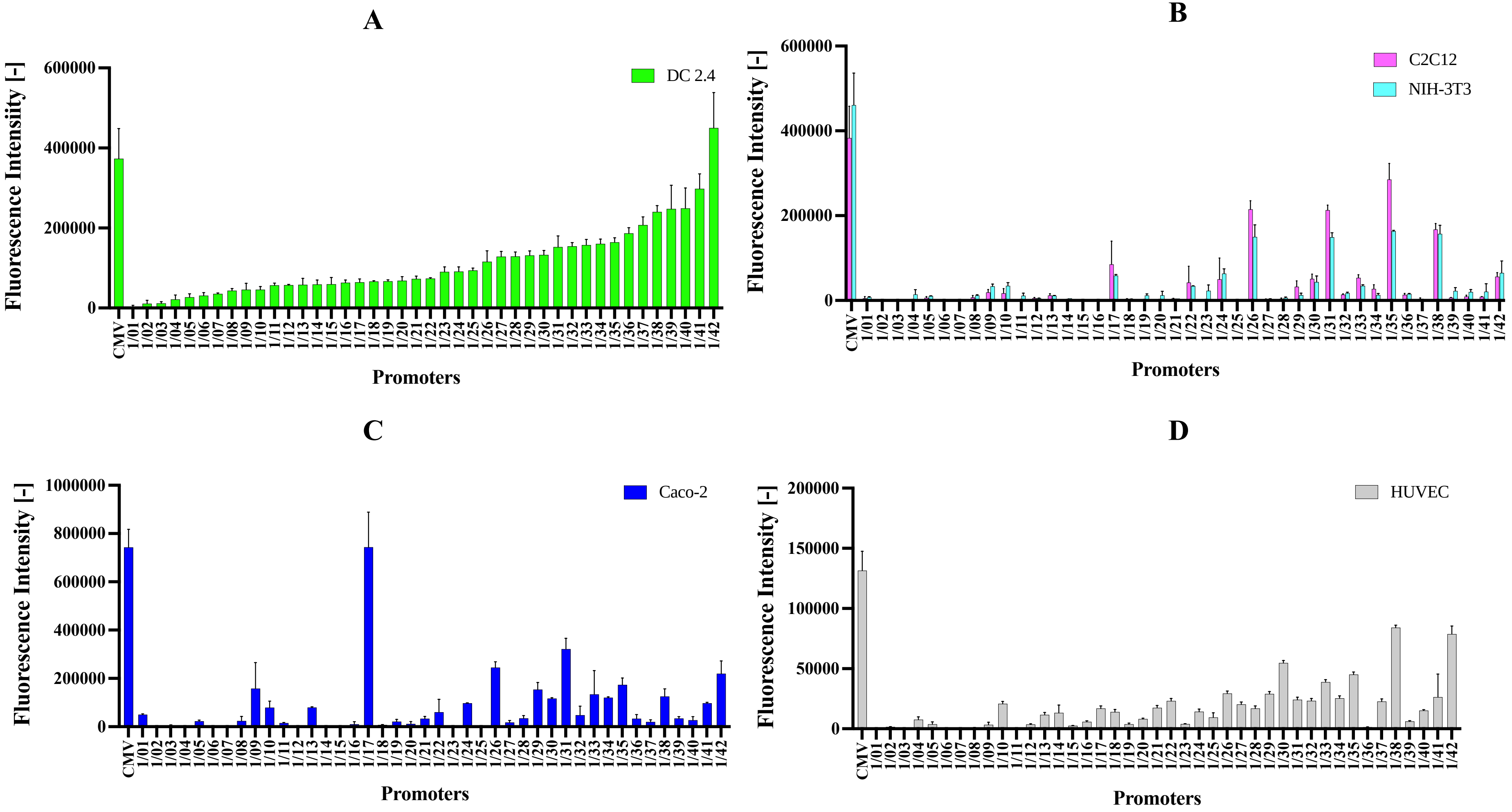

Figure S6: Specificity of dendritic cell-specific transcriptional activity across all 42 first library promoters visualised as the minimum fold change between the relative promoter activity in DC2.4 and the relative promoter activity across all off-target cells. 11 promoters (starred) exhibiting over 5-fold changes (marked by dotted line) are identified as dendritic cell-specific promoters.

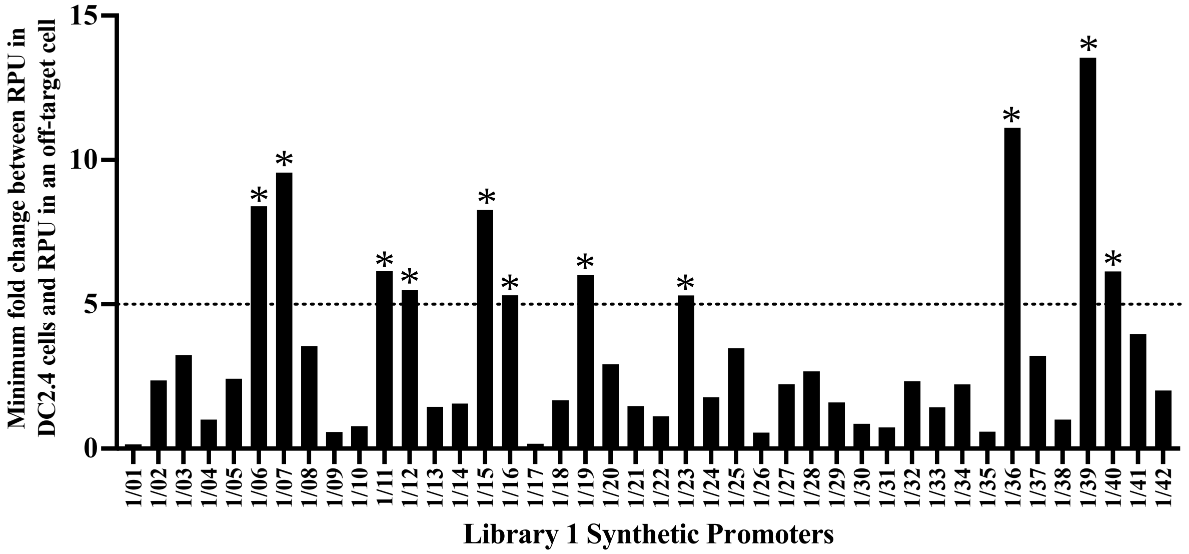

Figure S7: The dendritic cell-specific TFRE activity (DCSTA) score of all 12 TFREs arranged in descending order of magnitude.

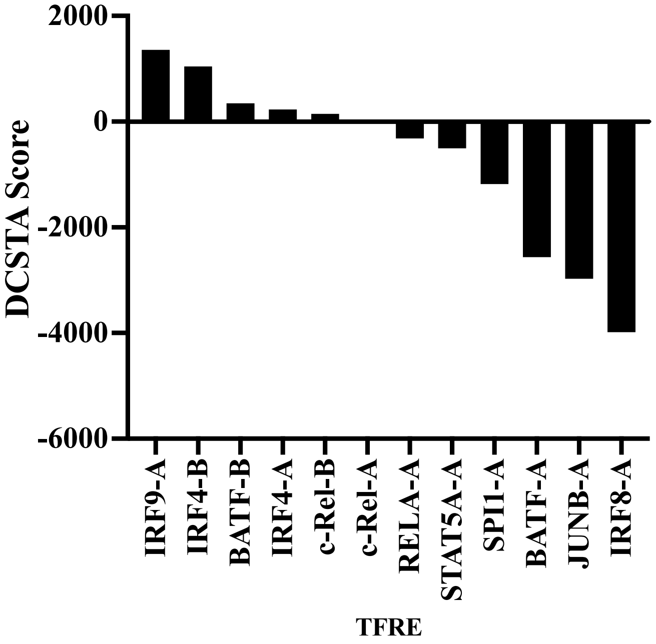

Table S11. TFRE composition, copy number of a second library of 16 dendritic cell-specific promoters.

Promoter	IRF9-A	RELA-A	c-Rel-A	SPI1-A	c-Rel-B	BATF-B	IRF4-B	IRF4-A	BATF-A
**2/01**	0	0	4	1	2	0	2	4	2
**2/02**	1	0	0	2	0	0	2	0	0
**2/03**	2	2	0	0	0	0	2	0	0
**2/04**	2	0	0	0	2	0	2	0	0
**2/05**	2	0	0	0	0	2	2	0	0
**2/06**	4	2	2	0	2	0	2	0	0
**2/07**	4	2	0	1	2	0	2	4	0
**2/08**	4	2	2	0	2	0	4	0	0
**2/09**	4	2	4	0	2	0	2	0	0
**2/10**	4	2	0	3	2	0	2	6	0
**2/11**	4	0	0	2	2	0	4	0	0
**2/12**	5	2	0	4	0	2	2	4	0
**2/13**	4	2	4	2	2	0	2	2	0
**2/14**	5	2	2	4	2	0	0	4	0
**2/15**	5	2	0	4	2	2	0	4	0
**2/16**	4	2	0	2	0	2	2	4	0

Figure S8: GFP fluorescence intensities of all 16 second library promoters and the hCMV-IE1 reference promoter (CMV) in DC 2.4, C2C12, NIH-3T3 and HUVEC cells. Values represent the mean ± standard deviation across three independent transfections each performed in triplicate.

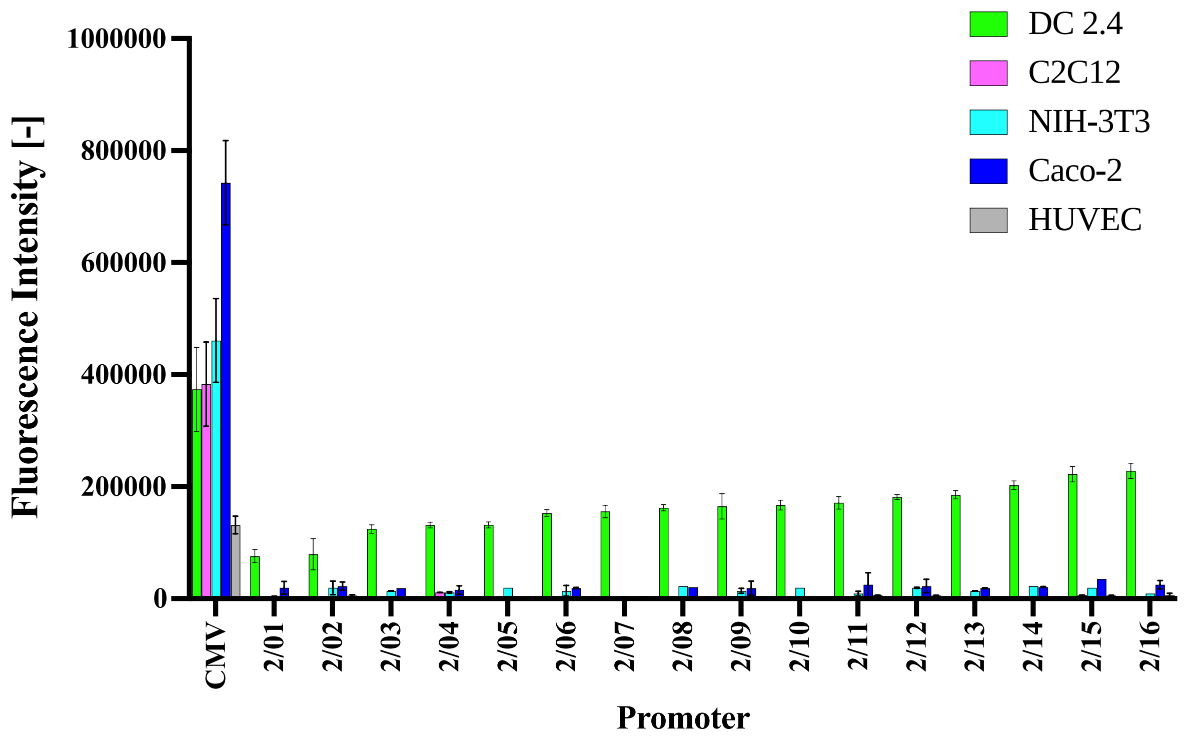

Figure S9. A simple linear regression of dendritic cell specific promoter activity score and the observed relative promoter activities of all 27 dendritic cell-specific promoters showing a strong positive relationship between both variables.

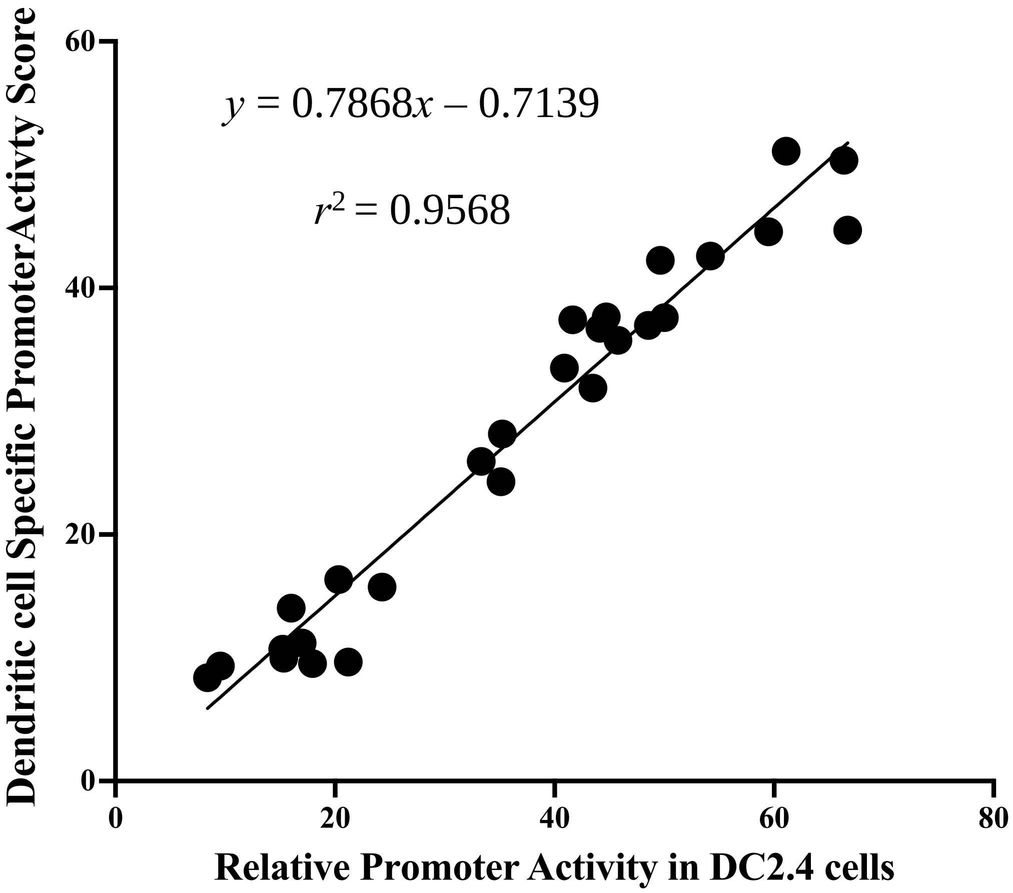

DC-SPECIFIC PROMOTER SEQUENCES

Colour code

TFRE	Colour
IRF9-A	Orange
IRF4-B	Blue
IRF4-A	Gold
SPI1-A	Purple
BATF-B	Blue-Grey
c-Rel-A	Grey
c-Rel-B	Light-Green
RELA-A	Red
BATF-A	Light-Blue
SPACERS	Black

FIRST LIBRARY

1. **1/36 – 471 bp**

CTAAACCGAGAATCGAAACTAAGCTCTAAACCGAGAATCGAAACTAAGCTATAGGAGGGCTA
AAGAAAGCAGAAAACTATAGGAGGGCTAAAGAAAGCAGAAACTAAACCGAGAATCGAAACTA
AGCTCTAAACCGAGAATCGAAACTAAGCTGAAGGACTTTCCAGCGAAGGACTTTCCAGCATA
GGAGGGCTAAAGAAAGCAGAAAACTATAGGAGGGCTAAAGAAAGCAGAAAGTGAAGGAGGAA
GTCTGAGGCTTGTGAAGGAGGAAGTCTGAGGCCTAAACCGAGAATCGAAACTAAGCTCTAAA
CCGAGAATCGAAACTAAGCTGTGAAGGAGGAAGTCTGAGGCTTGTGAAGGAGGAAGTCTGAG
GCATAGGAGGGCTAAAGAAAGCAGAAAACTATAGGAGGGCTAAAGAAAGCAGAAAGTGAAGG
AGGAAGTCTGAGGCTTGTGAAGGAGGAAGTCTGAGGC

---

2. **1/39 – 454 bp**

CTAAACCGAGAATCGAAACTAAGCTCTAAACCGAGAATCGAAACTAAGCTATAGGAGGGCTA
AAGAAAGCAGAAAACTATAGGAGGGCTAAAGAAAGCAGAAACTAAACCGAGAATCGAAACTA
AGCTCTAAACCGAGAATCGAAACTAAGCTGAAGGACTTTCCAGCGAAGGACTTTCCAGCCAG
GGTGGTTCCTTCCAGGGTGGTTCCTTCGTAGATGTGGAAGTGAAAGCTACAAGTAGATGTGG
AAGTGAAAGCTACAACTAAACCGAGAATCGAAACTAAGCTCTAAACCGAGAATCGAAACTAA
GCTGTGAAGGAGGAAGTCTGAGGCTTGTGAAGGAGGAAGTCTGAGGCATAGGAGGGCTAAAG
AAAGCAGAAAACTATAGGAGGGCTAAAGAAAGCAGAAAGTGAAGGAGGAAGTCTGAGGCTTG
TGAAGGAGGAAGTCTGAGGC

---

3. **1/15 – 177 bp**

CTTGGGGTTTCCAACCTTGGGGTTTCCAACGTAGATGTGGAAGTGAAAGCTACAAGTAGATG
TGGAAGTGAAAGCTACAAATAGGAGGGCTAAAGAAAGCAGAAAACTATAGGAGGGCTAAAGA
AAGCAGAAAGTGAAGGAGGAAGTCTGAGGCTTGTGAAGGAGGAAGTCTGAGGC

---

4. **1/40 – 274 bp**

CTAAACCGAGAATCGAAACTAAGCTCTAAACCGAGAATCGAAACTAAGCTGTAGATGTGGAA
GTGAAAGCTACAAGTAGATGTGGAAGTGAAAGCTACAACTAAACCGAGAATCGAAACTAAGC
TCTAAACCGAGAATCGAAACTAAGCTGAAGGACTTTCCAGCGAAGGACTTTCCAGCGTAGAT
GTGGAAGTGAAAGCTACAAGTAGATGTGGAAGTGAAAGCTACAAGTGAAGGAGGAAGTCTGA
GGCTTGTGAAGGAGGAAGTCTGAGGC

---

5. **1/06 – 374 bp**

CTTGGGGTTTCCAACCTTGGGGTTTCCAACGTAGATGTGGAAGTGAAAGCTACAAGTAGATG
TGGAAGTGAAAGCTACAAGTGAAGGAGGAAGTCTGAGGCTTGTGAAGGAGGAAGTCTGAGGC
GTAGATGTGGAAGTGAAAGCTACAAGTAGATGTGGAAGTGAAAGCTACAAGTGAAGGAGGAA
GTCTGAGGCTTGTGAAGGAGGAAGTCTGAGGCCTTGGGGTTTCCAACCTTGGGGTTTCCAAC
GTAGATGTGGAAGTGAAAGCTACAAGTAGATGTGGAAGTGAAAGCTACAACTTGGGGTTTCC
AACCTTGGGGTTTCCAACAAGTGAAGGAGGAAGTCTGAGGCTTGTGAAGGAGGAAGTCTGAG
GC

---

6. 1/16 - 600 bp

CTTGGGGTTTCCAACCTTGGGGTTTCCAACCTAAACCGAGAATCGAAACTAAGCTCTAAACC
GAGAATCGAAACTAAGCTCAGGGTGGTTCCTTCCAGGGTGGTTCCTTCCTAAACCGAGAATC
GAAACTAAGCTCTAAACCGAGAATCGAAACTAAGCTCTTGGGGTTTCCAACCTTGGGGTTTC
CAACGAAGGACTTTCCAGCGAAGGACTTTCCAGCGTAGATGTGGAAGTGAAAGCTACAAGTA
GATGTGGAAGTGAAAGCTACAACTTGGGGTTTCCAACCTTGGGGTTTCCAACTCTTGACTCA
GTCTCTTGACTCAGTCGTAGATGTGGAAGTGAAAGCTACAAGTAGATGTGGAAGTGAAAGCT
ACAACTAAACCGAGAATCGAAACTAAGCTCTAAACCGAGAATCGAAACTAAGCTGTAGATGT
GGAAGTGAAAGCTACAAGTAGATGTGGAAGTGAAAGCTACAAGTGAAGGAGGAAGTCTGAGG
CTTGTGAAGGAGGAAGTCTGAGGCCTTGGGGTTTCCAACCTTGGGGTTTCCAACGTAGATGT
GGAAGTGAAAGCTACAAGTAGATGTGGAAGTGAAAGCTACAA

7. 1/07 - 400 bp

CTTGGGGTTTCCAACCTTGGGGTTTCCAACGTAGATGTGGAAGTGAAAGCTACAAGTAGATG
TGGAAGTGAAAGCTACAACTTGGGGTTTCCAACCTTGGGGTTTCCAACTCTTGACTCAGTCT
CTTGACTCAGTCGTAGATGTGGAAGTGAAAGCTACAAGTAGATGTGGAAGTGAAAGCTACAA
GTGAAGGAGGAAGTCTGAGGCTTGTGAAGGAGGAAGTCTGAGGCCTTGGGGTTTCCAACCTT
GGGGTTTCCAACTTGTGAAGGAGGAAGTCTGAGGCTTGTGAAGGAGGAAGTCTGAGGCGTAG
ATGTGGAAGTGAAAGCTACAAGTAGATGTGGAAGTGAAAGCTACAAGTGAAGGAGGAAGTCT
GAGGCTTGTGAAGGAGGAAGTCTGAGGC

8. 1/11 - 303 bp

GTAGATGTGGAAGTGAAAGCTACAAGTAGATGTGGAAGTGAAAGCTACAACAGGGTGGTTCC
TTCCAGGGTGGTTCCTTCGTAGATGTGGAAGTGAAAGCTACAAGTAGATGTGGAAGTGAAAG
CTACAATCTTGACTCAGTCTCTTGACTCAGTCGTAGATGTGGAAGTGAAAGCTACAAGTAGA
TGTGGAAGTGAAAGCTACAAATAGGAGGGCTAAAGAAAGCAGAAAACTATAGGAGGGCTAAA
GAAAGCAGAAAGTGAAGGAGGAAGTCTGAGGCTTGTGAAGGAGGAAGTCTGAGGC

9. 1/12 - 318 bp

CTTGGGGTTTCCAACCTTGGGGTTTCCAACGTAGATGTGGAAGTGAAAGCTACAAGTAGATG
TGGAAGTGAAAGCTACAATCTTGACTCAGTCTCTTGACTCAGTCCTTGGGGTTTCCAACCTT
GGGGTTTCCAACTCTTGACTCAGTCTCTTGACTCAGTCGTAGATGTGGAAGTGAAAGCTACA
AGTAGATGTGGAAGTGAAAGCTACAATCTTGACTCAGTCTCTTGACTCAGTCCTTGGGGTTT
CCAACCTTGGGGTTTCCAACGTAGATGTGGAAGTGAAAGCTACAAGTAGATGTGGAAGTGAA
AGCTACAA

10. 1/23 - 451 bp

CTAAACCGAGAATCGAAACTAAGCTCTAAACCGAGAATCGAAACTAAGCTGTAGATGTGGAA
GTGAAAGCTACAAGTAGATGTGGAAGTGAAAGCTACAACTAAACCGAGAATCGAAACTAAGC
TCTAAACCGAGAATCGAAACTAAGCTGAAGGACTTTCCAGCGAAGGACTTTCCAGCGTAGAT
GTGGAAGTGAAAGCTACAAGTAGATGTGGAAGTGAAAGCTACAACTTGGGGTTTCCAACCTT
GGGGTTTCCAACGTAGATGTGGAAGTGAAAGCTACAAGTAGATGTGGAAGTGAAAGCTACAA
GTGAAGGAGGAAGTCTGAGGCTTGTGAAGGAGGAAGTCTGAGGCATAGGAGGGCTAAAGAAA
GCAGAAAACTATAGGAGGGCTAAAGAAAGCAGAAAGTGAAGGAGGAAGTCTGAGGCTTGTGA
AGGAGGAAGTCTGAGGC

11. 1/19 - 449 bp

CTAAACCGAGAATCGAAACTAAGCTCTAAACCGAGAATCGAAACTAAGCTGTAGATGTGGAA
GTGAAAGCTACAAGTAGATGTGGAAGTGAAAGCTACAACTAAACCGAGAATCGAAACTAAGC
TCTAAACCGAGAATCGAAACTAAGCTACTCTTGACTCAGTCTCTTGACTCAGTCGTAGATGT
GGAAGTGAAAGCTACAAGTAGATGTGGAAGTGAAAGCTACAACTTGGGGTTTCCAACCTTGG
GGTTTCCAACGTAGATGTGGAAGTGAAAGCTACAAGTAGATGTGGAAGTGAAAGCTACAAGT
GAAGGAGGAAGTCTGAGGCTTGTGAAGGAGGAAGTCTGAGGCATAGGAGGGCTAAAGAAAGC
AGAAAACTATAGGAGGGCTAAAGAAAGCAGAAAGTGAAGGAGGAAGTCTGAGGCTTGTGAAG
GAGGAAGTCTGAGGC

SECOND LIBRARY

---

12. 2/13 – 367 bp

CTTGGGGTTTCCAACCTTGGGGTTTCCAACCTAAACCGAGAATCGAAACTAAGCTCTAAACC
GAGAATCGAAACTAAGCTCAGGGTGGTTCCTTCCAGGGTGGTTCCTTCCTAAACCGAGAATC
GAAACTAAGCTCTAAACCGAGAATCGAAACTAAGCTCTTGGGGTTTCCAACCTTGGGGTTTC
CAACGAAGGACTTTCCAGCGAAGGACTTTCCAGCGTAGATGTGGAAGTGAAAGCTACAAGTA
GATGTGGAAGTGAAAGCTACAAATAGGAGGGCTAAAGAAAGCAGAAAACTATAGGAGGGCTA
AAGAAAGCAGAAAGTGAAGGAGGAAGTCTGAGGCTTGTGAAGGAGGAAGTCTGAGGC

---

13. 2/01 - 295 bp

CTTGGGGTTTCCAACCTTGGGGTTTCCAACATAGGAGGGCTAAAGAAAGCAGAAAACTATAG
GAGGGCTAAAGAAAGCAGAAACTTGGGGTTTCCAACCTTGGGGTTTCCAACTCCTGACTCAC
TGTATCCTGACTCACTGATAGGAGGGCTAAAGAAAGCAGAAAACTATAGGAGGGCTAAAGAA
AGCAGAAAGTGAAGGAGGAAGTCTGAGGCCAGGGTGGTTCCTTCCAGGGTGGTTCCTTCGTA
GATGTGGAAGTGAAAGCTACAAGTAGATGTGGAAGTGAAAGCTACAA

---

14. 2/04 - 130 bp

CTAAACCGAGAATCGAAACTAAGCTCTAAACCGAGAATCGAAACTAAGCTCAGGGTGGTTCC
TTCCAGGGTGGTTCCTTCGTAGATGTGGAAGTGAAAGCTACAAGTAGATGTGGAAGTGAAAG
CTACAA

---

15. 2/07 - 337 bp

CTAAACCGAGAATCGAAACTAAGCTCTAAACCGAGAATCGAAACTAAGCTATAGGAGGGCTA
AAGAAAGCAGAAAACTATAGGAGGGCTAAAGAAAGCAGAAACTAAACCGAGAATCGAAACTA
AGCTCTAAACCGAGAATCGAAACTAAGCTGAAGGACTTTCCAGCGAAGGACTTTCCAGCATA
GGAGGGCTAAAGAAAGCAGAAAACTATAGGAGGGCTAAAGAAAGCAGAAAGTGAAGGAGGAA
GTCTGAGGCCAGGGTGGTTCCTTCCAGGGTGGTTCCTTCGTAGATGTGGAAGTGAAAGCTAC
AAGTAGATGTGGAAGTGAAAGCTACAA

---

16. 2/10 - 434 bp

CTAAACCGAGAATCGAAACTAAGCTCTAAACCGAGAATCGAAACTAAGCTATAGGAGGGCTA
AAGAAAGCAGAAAACTATAGGAGGGCTAAAGAAAGCAGAAACTAAACCGAGAATCGAAACTA
AGCTCTAAACCGAGAATCGAAACTAAGCTGAAGGACTTTCCAGCGAAGGACTTTCCAGCATA
GGAGGGCTAAAGAAAGCAGAAAACTATAGGAGGGCTAAAGAAAGCAGAAAGTGAAGGAGGAA
GTCTGAGGCCAGGGTGGTTCCTTCCAGGGTGGTTCCTTCGTAGATGTGGAAGTGAAAGCTAC
AAGTAGATGTGGAAGTGAAAGCTACAAATAGGAGGGCTAAAGAAAGCAGAAAACTATAGGAG
GGCTAAAGAAAGCAGAAAGTGAAGGAGGAAGTCTGAGGCTTGTGAAGGAGGAAGTCTGAGGC

---

17. 2/16 - 356 bp

CTAAACCGAGAATCGAAACTAAGCTCTAAACCGAGAATCGAAACTAAGCTATAGGAGGGCTA
AAGAAAGCAGAAAACTATAGGAGGGCTAAAGAAAGCAGAAACTAAACCGAGAATCGAAACTA
AGCTCTAAACCGAGAATCGAAACTAAGCTGAAGGACTTTCCAGCGAAGGACTTTCCAGCTCT
TGACTCAGTCTCTTGACTCAGTCGTAGATGTGGAAGTGAAAGCTACAAGTAGATGTGGAAGT

GAAAGCTACAAATAGGAGGGCTAAAGAAAGCAGAAAACTATAGGAGGGCTAAAGAAAGCAGA
AAGTGAAGGAGGAAGTCTGAGGCTTGTGAAGGAGGAAGTCTGAGGC

---

18. 2/12 - 425 bp

ctaaaccgagaatcgaaactaagctctaaaccgagaatcgaaactaagctataggagggcta
aaGAAAgcagaaaACTataggagggctaaaGAAAgcagaaactaaaccgagaatcgaaacta
agctctaaaccgagaatcgaaactaagctgaaggactttccAGCGAAGGACTTTCCAGCtct
tgactcagtctcttgactcagtcgtagatgtggaagtgaaagctacaagtagatgtggaagt
gaaagctacaaCTAAACCGAGAATCGAAACTAAGctgtgaaggaggaagtctgaggcTTgtg
aaggaggaagtctgaggcataggagggctaaaGAAAgcagaaaACTataggagggctaaaGA
AAgcagaaagtgaaggaggaagtctgaggcTTgtgaaggaggaagtctgaggc

---

19. 2/09 - 270 bp

CTTGGGGTTTCCAACCTTGGGGTTTCCAACCTAAACCGAGAATCGAAACTAAGCTCTAAACC
GAGAATCGAAACTAAGCTCAGGGTGGTTCCTTCCAGGGTGGTTCCTTCCTAAACCGAGAATC
GAAACTAAGCTCTAAACCGAGAATCGAAACTAAGCTCTTGGGGTTTCCAACCTTGGGGTTTC
CAACGAAGGACTTTCCAGCGAAGGACTTTCCAGCGTAGATGTGGAAGTGAAAGCTACAAGTA
GATGTGGAAGTGAAAGCTACAA

---

20. 2/06 – 240 bp

CTAAACCGAGAATCGAAACTAAGCTCTAAACCGAGAATCGAAACTAAGCTCAGGGTGGTTCC
TTCCAGGGTGGTTCCTTCCTAAACCGAGAATCGAAACTAAGCTCTAAACCGAGAATCGAAAC
TAAGCTCTTGGGGTTTCCAACCTTGGGGTTTCCAACGAAGGACTTTCCAGCGAAGGACTTTC
CAGCGTAGATGTGGAAGTGAAAGCTACAAGTAGATGTGGAAGTGAAAGCTACAA

---

21. 2/05 – 126 bp

CTAAACCGAGAATCGAAACTAAGCTCTAAACCGAGAATCGAAACTAAGCTTCTTGACTCAGT
CTCTTGACTCAGTCGTAGATGTGGAAGTGAAAGCTACAAGTAGATGTGGAAGTGAAAGCTAC
AA

---

22. 2/02 – 119 bp

CTAAACCGAGAATCGAAACTAAGCTGTGAAGGAGGAAGTCTGAGGCTTGTGAAGGAGGAAGT
CTGAGGCGTAGATGTGGAAGTGAAAGCTACAAGTAGATGTGGAAGTGAAAGCTACAA

---

23. 2/03 – 130 bp

CTAAACCGAGAATCGAAACTAAGCTCTAAACCGAGAATCGAAACTAAGCTGAAGGACTTTCC
AGCGAAGGACTTTCCAGCGTAGATGTGGAAGTGAAAGCTACAAGTAGATGTGGAAGTGAAAG
CTACAA

---

24. 2/11 - 280 bp

CTAAACCGAGAATCGAAACTAAGCTCTAAACCGAGAATCGAAACTAAGCTGTGAAGGAGGAA
GTCTGAGGCTTGTGAAGGAGGAAGTCTGAGGCGTAGATGTGGAAGTGAAAGCTACAAGTAGA
TGTGGAAGTGAAAGCTACAAACGCGTCTAAACCGAGAATCGAAACTAAGCTCTAAACCGAGA

ATCGAAACTAAGCTCAGGGTGGTTCCTTCCAGGGTGGTTCCTTCGTAGATGTGGAAGTGAAA
GCTACAAGTAGATGTGGAAGTGAAAGCTACAA

---

25. 2/08 - 296 bp

CTAAACCGAGAATCGAAACTAAGCTCTAAACCGAGAATCGAAACTAAGCTGAAGGACTTTCC
AGCGAAGGACTTTCCAGCCTTGGGGTTTCCAACCTTGGGGTTTCCAACGTAGATGTGGAAGT
GAAAGCTACAAGTAGATGTGGAAGTGAAAGCTACAAACGCGTCTAAACCGAGAATCGAAACT
AAGCTCTAAACCGAGAATCGAAACTAAGCTCAGGGTGGTTCCTTCCAGGGTGGTTCCTTCGT
AGATGTGGAAGTGAAAGCTACAAGTAGATGTGGAAGTGAAAGCTACAA

---

26. 2/15 - 405 bp

CTAAACCGAGAATCGAAACTAAGCTCTAAACCGAGAATCGAAACTAAGCTATAGGAGGGCTA
AAGAAAGCAGAAAACTATAGGAGGGCTAAAGAAAGCAGAAACTAAACCGAGAATCGAAACTA
AGCTCTAAACCGAGAATCGAAACTAAGCTGAAGGACTTTCCAGCGAAGGACTTTCCAGCCAG
GGTGGTTCCTTCCAGGGTGGTTCCTTCTCTTGACTCAGTCTCTTGACTCAGTCCTAAACCGA
GAATCGAAACTAAGCTGTGAAGGAGGAAGTCTGAGGCTTGTGAAGGAGGAAGTCTGAGGCAT
AGGAGGGCTAAAGAAAGCAGAAAACTATAGGAGGGCTAAAGAAAGCAGAAAGTGAAGGAGGA
AGTCTGAGGCTTGTGAAGGAGGAAGTCTGAGGC

---

27. 2/14 - 409 bp

CTAAACCGAGAATCGAAACTAAGCTCTAAACCGAGAATCGAAACTAAGCTATAGGAGGGCTA
AAGAAAGCAGAAAACTATAGGAGGGCTAAAGAAAGCAGAAACTAAACCGAGAATCGAAACTA
AGCTCTAAACCGAGAATCGAAACTAAGCTGAAGGACTTTCCAGCGAAGGACTTTCCAGCCTT
GGGGTTTCCAACCTTGGGGTTTCCAACCAGGGTGGTTCCTTCCAGGGTGGTTCCTTCCTAAA
CCGAGAATCGAAACTAAGCTGTGAAGGAGGAAGTCTGAGGCTTGTGAAGGAGGAAGTCTGAG
GCATAGGAGGGCTAAAGAAAGCAGAAAACTATAGGAGGGCTAAAGAAAGCAGAAAGTGAAGG
AGGAAGTCTGAGGCTTGTGAAGGAGGAAGTCTGAGGC

REFERENCES

[1] Sun, X. M., Hua, S., Chen, H. R., Ouyang, Z. Y., Einkauf, K., Tse, S., Ard, K., Ciaranello, A., Yawetz, S., Sax, P., Rosenberg, E. S., Lichterfeld, M., and Yu, X. G. (2017) Transcriptional Changes during Naturally Acquired Zika Virus Infection Render Dendritic Cells Highly Conducive to Viral Replication, *Cell Rep. 21*, 3471-3482.

[2] Hruz, T., Laule, O., Szabo, G., Wessendorp, F., Bleuler, S., Oertle, L., Widmayer, P., Gruissem, W., and Zimmermann, P. (2008) Genevestigator V3: A Reference Expression Database for the Meta-Analysis of Transcriptomes, *Adv. Bioinf. 2008*, 1-5.

[3] Manh, T. P. V., Alexandre, Y., Baranek, T., Crozat, K., and Dalod, M. (2013) Plasmacytoid, conventional, and monocyte-derived dendritic cells undergo a profound and convergent genetic reprogramming during their maturation, *Eur. J. Immunol. 43*, 1706-1715.
